# Supplementary material for: Mapping the S1 and S1’ subsites of cysteine proteases with new dipeptidyl nitrile inhibitors as trypanocidal agents
Source: PLoS Negl Trop Dis. 2020 Mar 12;14(3):e0007755. doi: 10.1371/journal.pntd.0007755 (PMC7067379; doi:10.1371/journal.pntd.0007755)
Supplement: S1 Fig — Plot of pKi (Cz) vs. pKi (LmCPB). A linear trendline fitted points. (PDF) [file pntd.0007755.s001.pdf]

Plot of pK_i (Cz) vs. pK_i (LmCPB). A linear trendline fitted points.

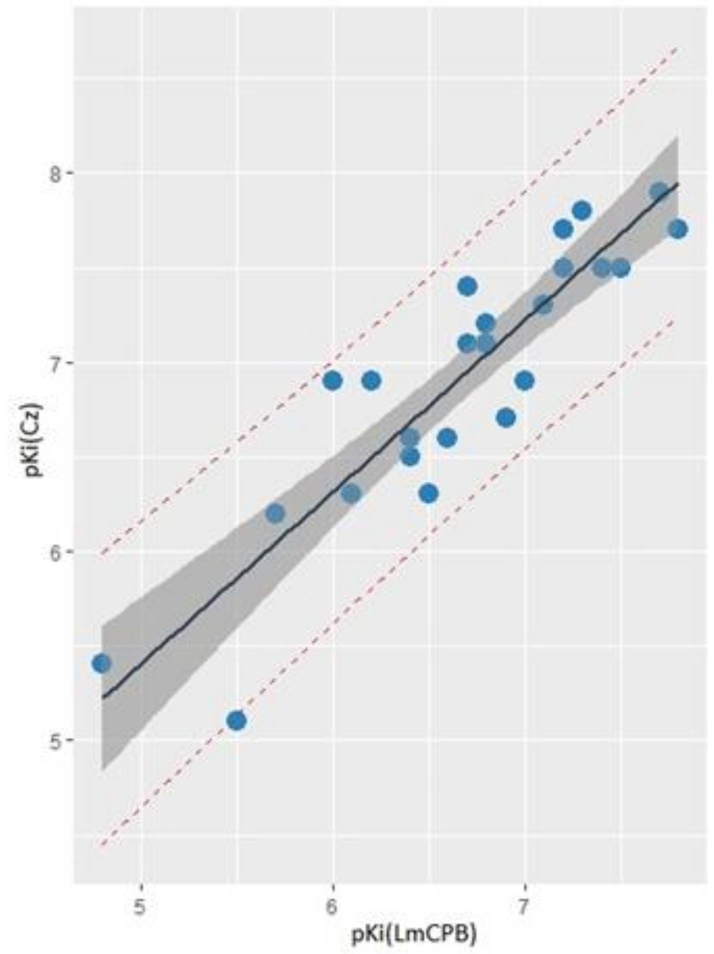

Multiple R^2 = 0.809; Adjusted R^2 = 0.800. F-statistic: 97.2 on 1 and 23 DF; p-value: $1 \times e^{-09}$.

Gray hatch: 95% confidence limit; Dashed red line: prediction interval
