# Supplementary material for: Mapping the S1 and S1’ subsites of cysteine proteases with new dipeptidyl nitrile inhibitors as trypanocidal agents
Source: PLoS Negl Trop Dis. 2020 Mar 12;14(3):e0007755. doi: 10.1371/journal.pntd.0007755 (PMC7067379; doi:10.1371/journal.pntd.0007755)
Supplement: S2 Fig — Dose curve response for determination of CC50 (LLCMK2) and EC50 (T. cruzi Tulahuen) for all compounds. (PDF) [file pntd.0007755.s002.pdf]

Figure S1. Dose curve response for determination of CC_{50} (LLCMK2) and EC_{50} (T. cruzi Tulahuen) for all compounds.

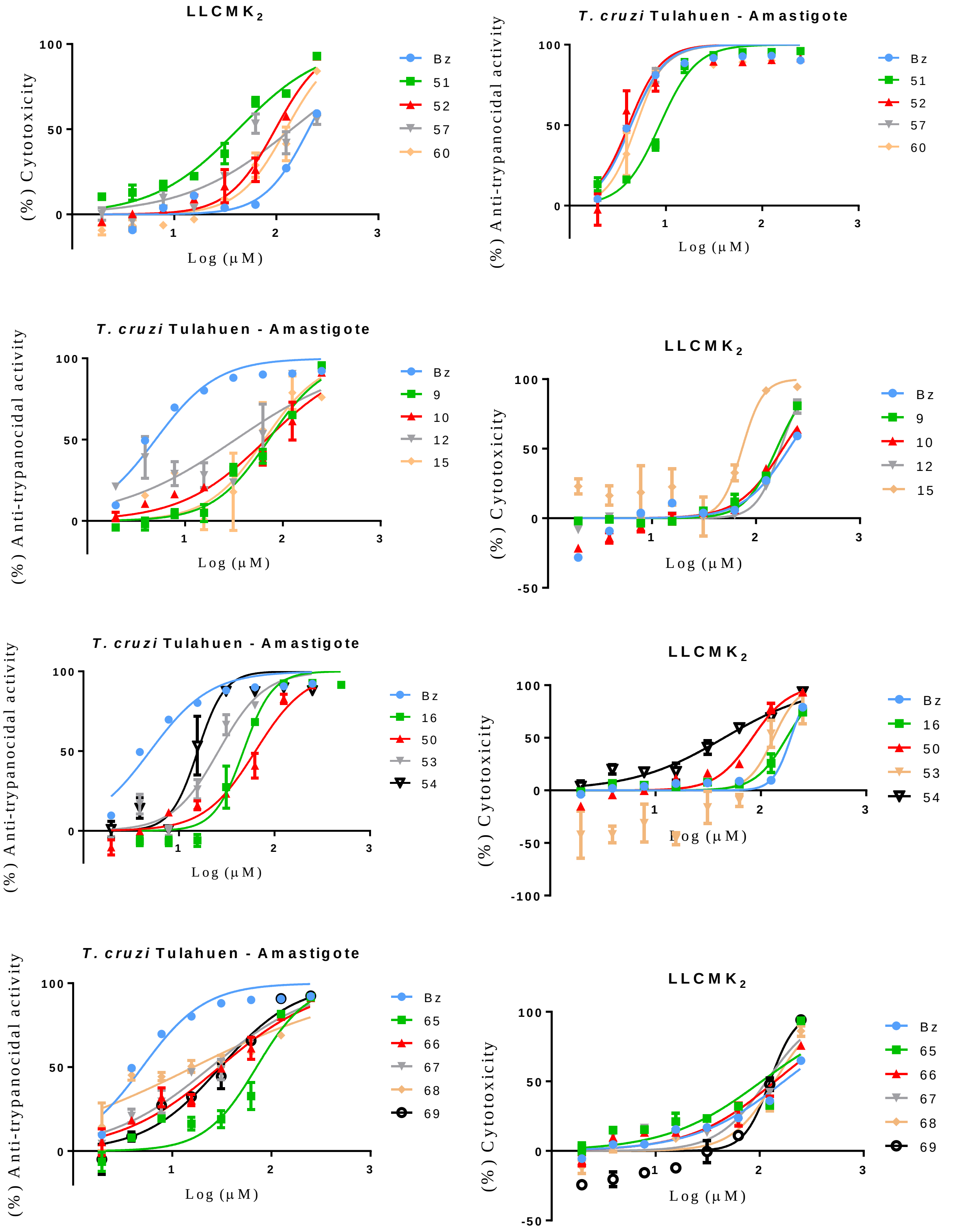

All experiments were at least performed in duplicate.
