# Supplementary material for: Mapping the S1 and S1’ subsites of cysteine proteases with new dipeptidyl nitrile inhibitors as trypanocidal agents
Source: PLoS Negl Trop Dis. 2020 Mar 12;14(3):e0007755. doi: 10.1371/journal.pntd.0007755 (PMC7067379; doi:10.1371/journal.pntd.0007755)
Supplement: S3 Fig — 1H, 13C NMR spectra and HPLC reports for final compounds. (PDF) [file pntd.0007755.s003.pdf]

^{1}H, ^{13}C NMR Spectra and HPLC Reports for Final Compounds.

*(S)-N-(3-(3-chlorophenyl)-1-((1-cyanocyclopropyl)amino)-1-oxopropan-2-yl)benzamide (**7**)*

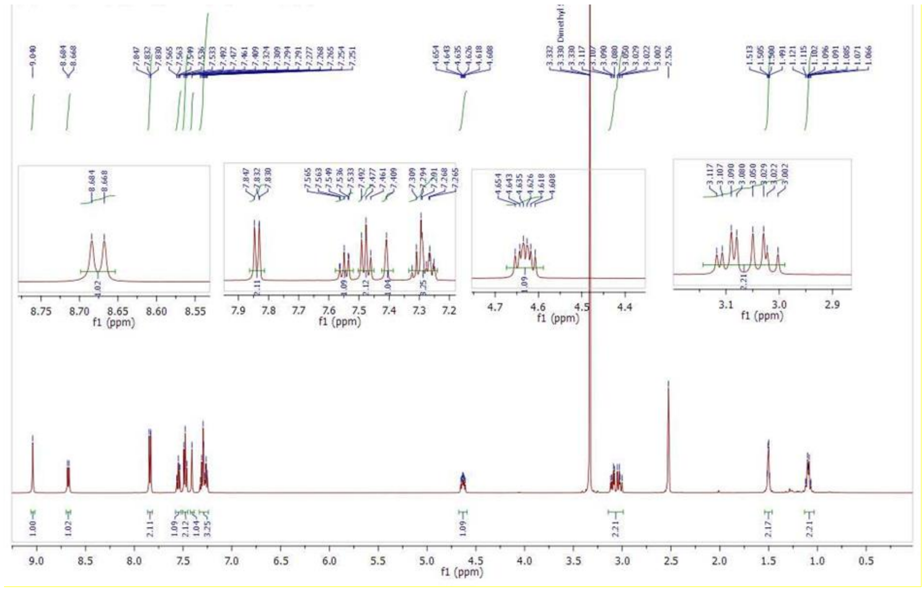

*Figure S1. ^{1}H NMR (500 MHz, DMSO_d_6) of compound **7**.*

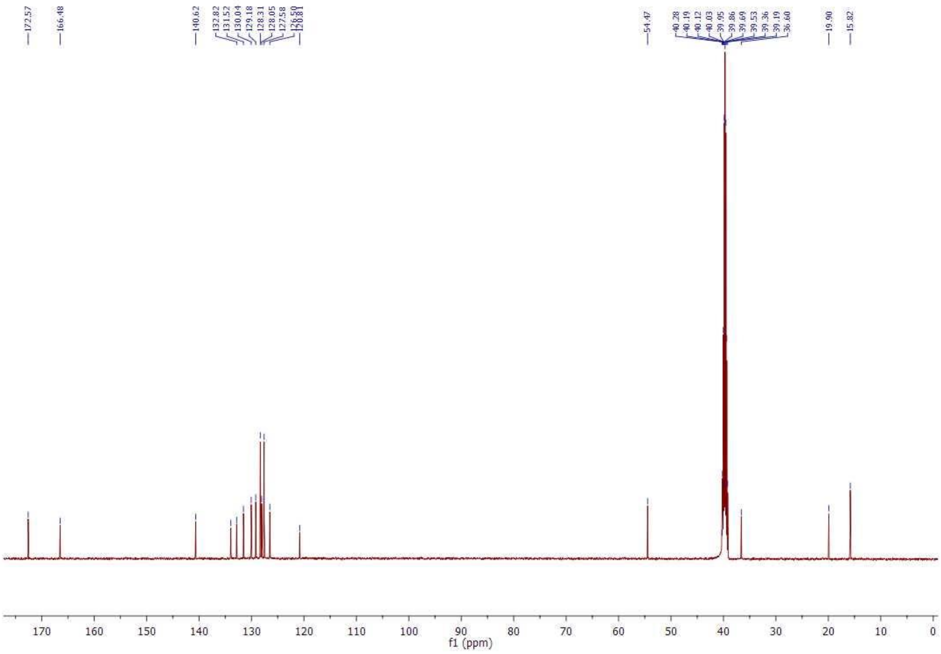

*Figure S2. ^{13}C NMR (125 MHz, DMSO_d_6) of compound **7**.*

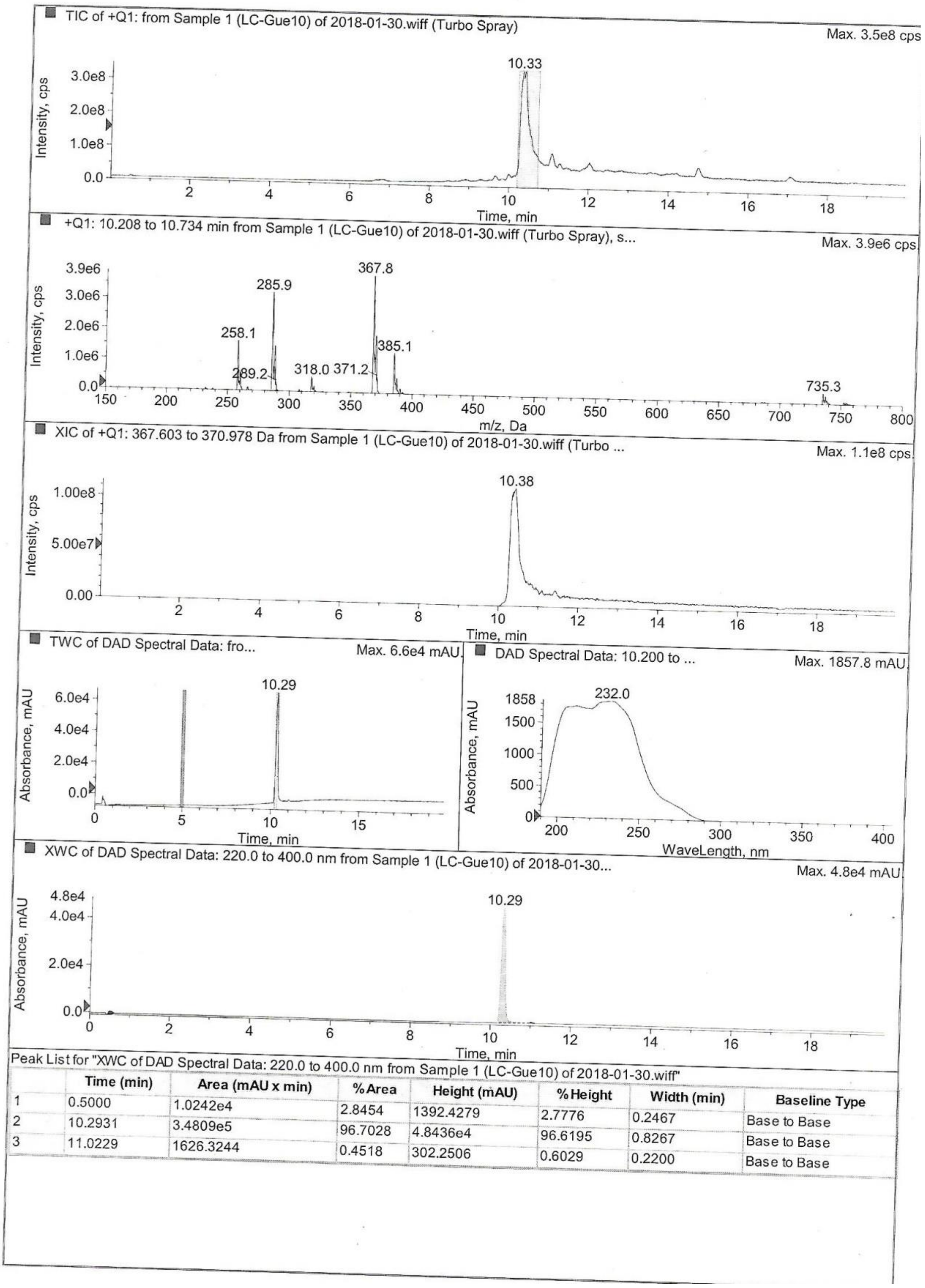

Peak List for "XWC of DAD Spectral Data: 220.0 to 400.0 nm from Sample 1 (LC-Gue10) of 2018-01-30.wiff"

	Time (min)	Area (mAU x min)	%Area	Height (mAU)	%Height	Width (min)	Baseline Type
1	0.5000	1.0242e4	2.8454	1392.4279	2.7776	0.2467	Base to Base
2	10.2931	3.4809e5	96.7028	4.8436e4	96.6195	0.8267	Base to Base
3	11.0229	1626.3244	0.4518	302.2506	0.6029	0.2200	Base to Base

*Figure S3. LC-MS report for compound **7**.*

*(S)-3-(tert-butyl)-N-(3-(3-chlorophenyl)-1-((1-cyanocyclopropyl)amino)-1-oxopropan-2-yl)-1-methyl-1H-pyrazole-5-carboxamide (**8**)*

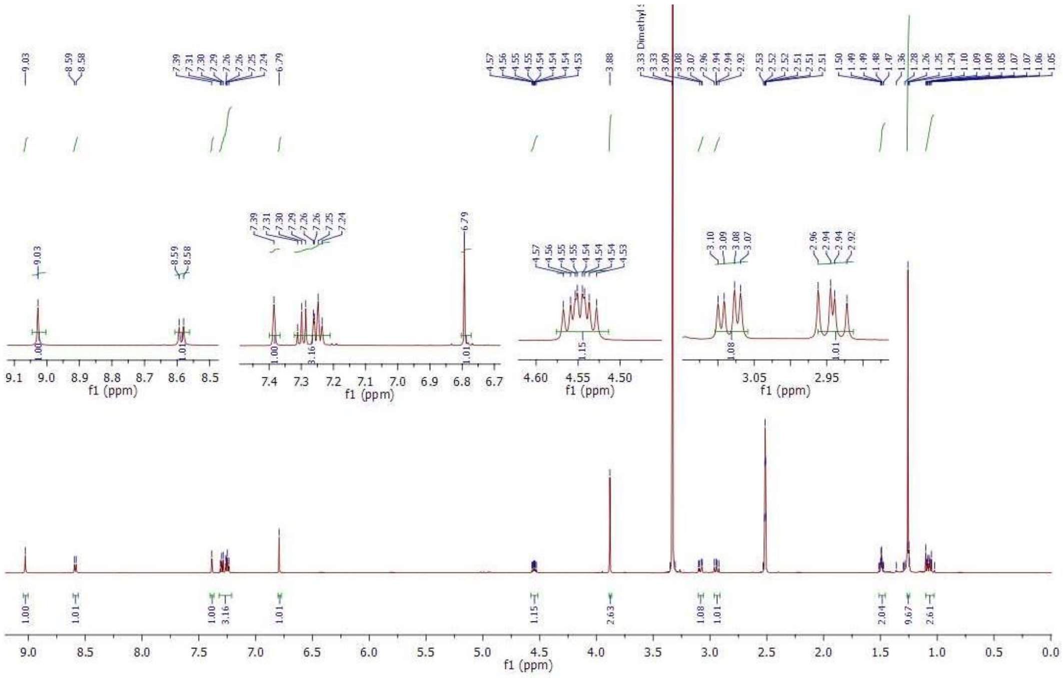

*Figure S4. ^{1}H NMR (500 MHz, DMSO_d_6) of compound **8**.*

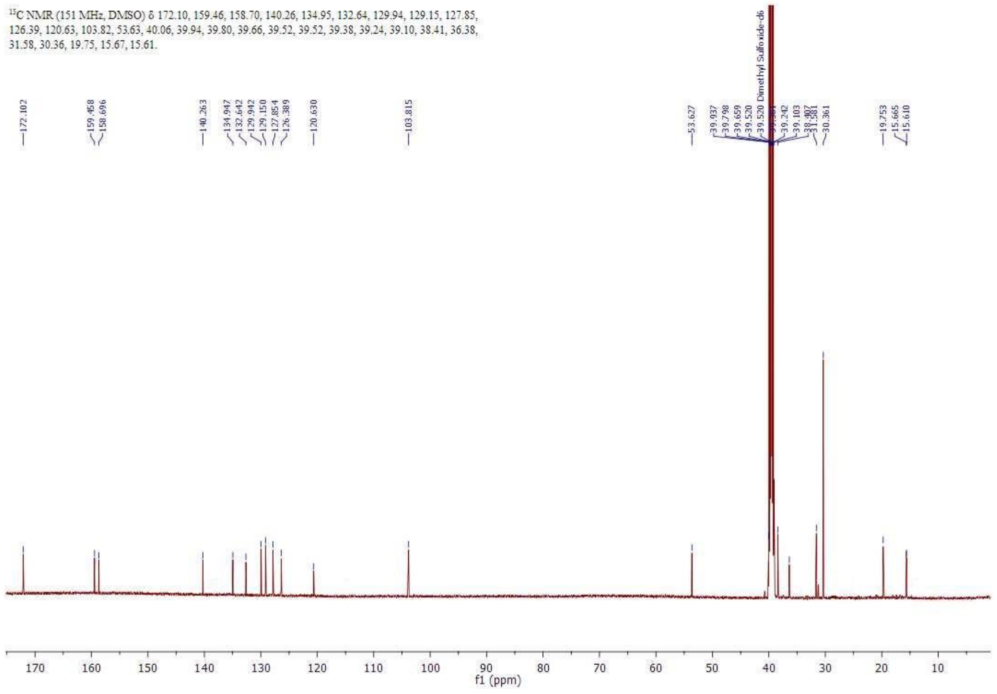

*Figure S5. ^{13}C NMR (125 MHz, DMSO_d_6) of compound **8**.*

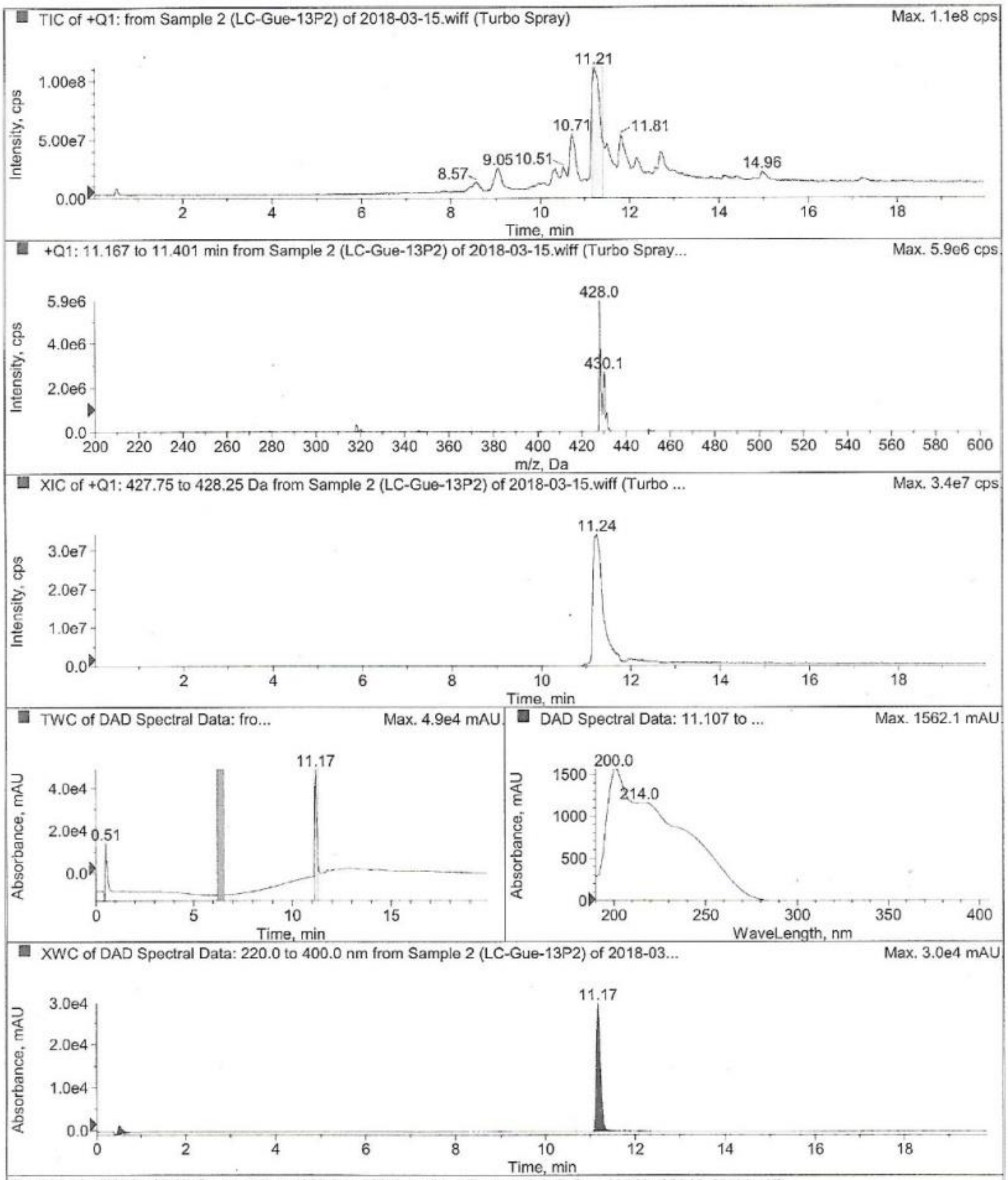

Peak List for "XWC of DAD Spectral Data: 220.0 to 400.0 nm from Sample 2 (LC-Gue-13P2) of 2018-03-15.wiff"

	Time (min)	Area (mAU x min)	%Area	Height (mAU)	%Height	Width (min)	Baseline Type
1	0.5111	1.3123e4	6.3512	2002.9344	6.2111	0.3200	Base to Base
2	8.4944	252.6088	0.1223	30.3513	0.0941	0.3600	Base to Base
3	8.9813	679.8270	0.3290	83.7299	0.2596	0.3333	Base to Base
4	10.6542	700.2268	0.3389	102.1618	0.3168	0.3200	Base to Base
5	11.1744	1.8948e5	91.7068	2.9652e4	91.9519	0.5867	Base to Base
6	11.7669	2163.2622	1.0470	335.5000	1.0404	0.3333	Base to Base
7	12.1120	216.4977	0.1048	40.6433	0.1260	0.1800	Base to Base

*Figure S6. LC-MS report for compound **8**.*

*(S)-3-(tert-butyl)-N-(1-((1-cyanocyclopropyl)amino)-1-oxo-3-(pyridin-4-yl)propan-2-yl)-1-methyl-1H-pyrazole-5-carboxamide (**10**)*

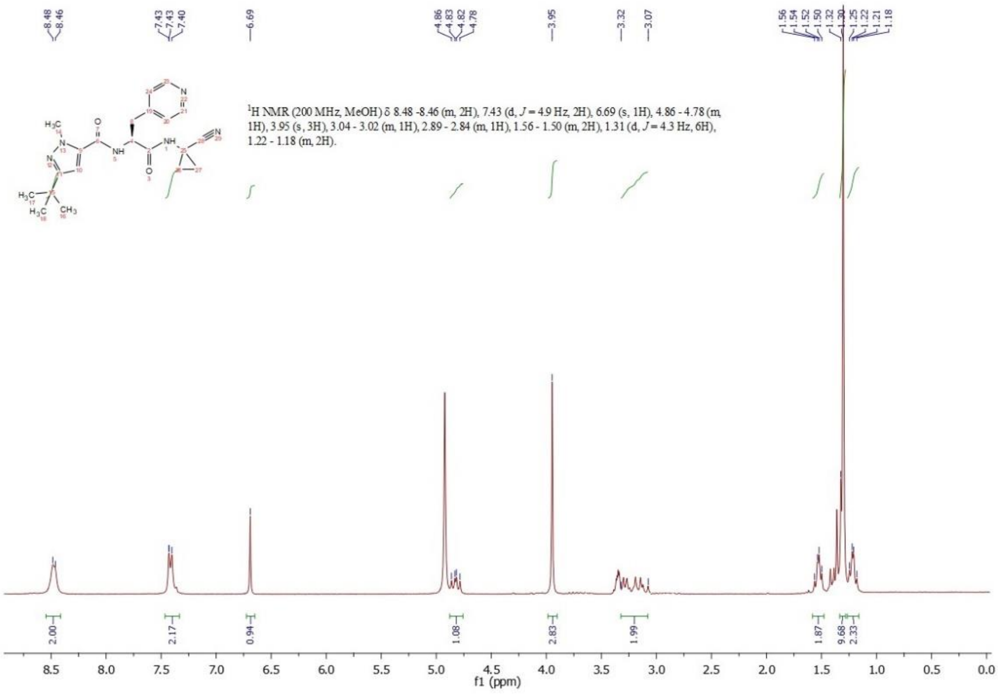

*Figure S7. ^{1}H NMR (200 MHz, CD_3OD) of compound **10**.*

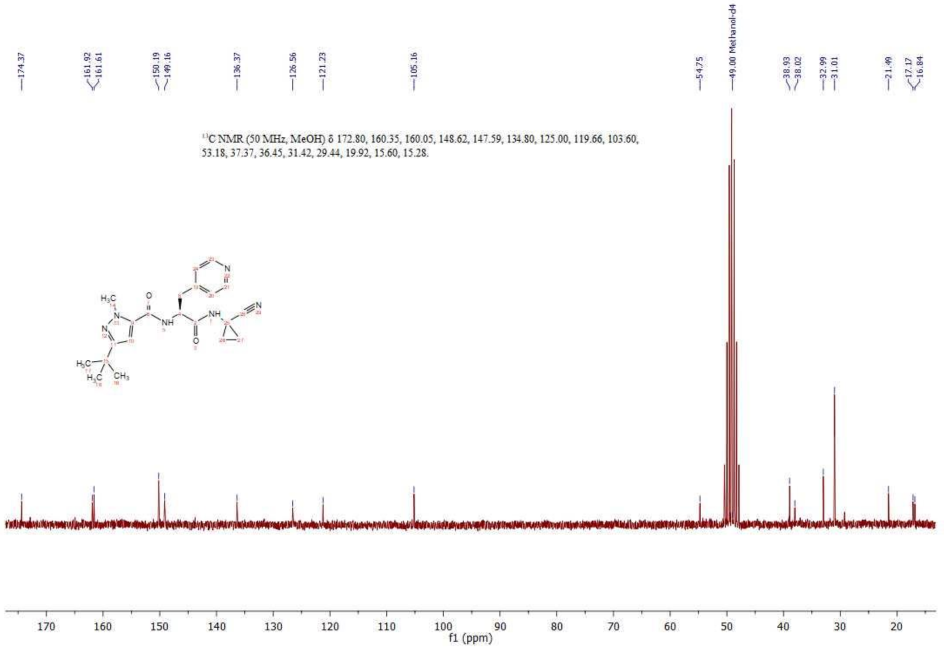

*Figure S8. ^{13}C NMR (50 MHz, CD_3OD) of compound **10**.*

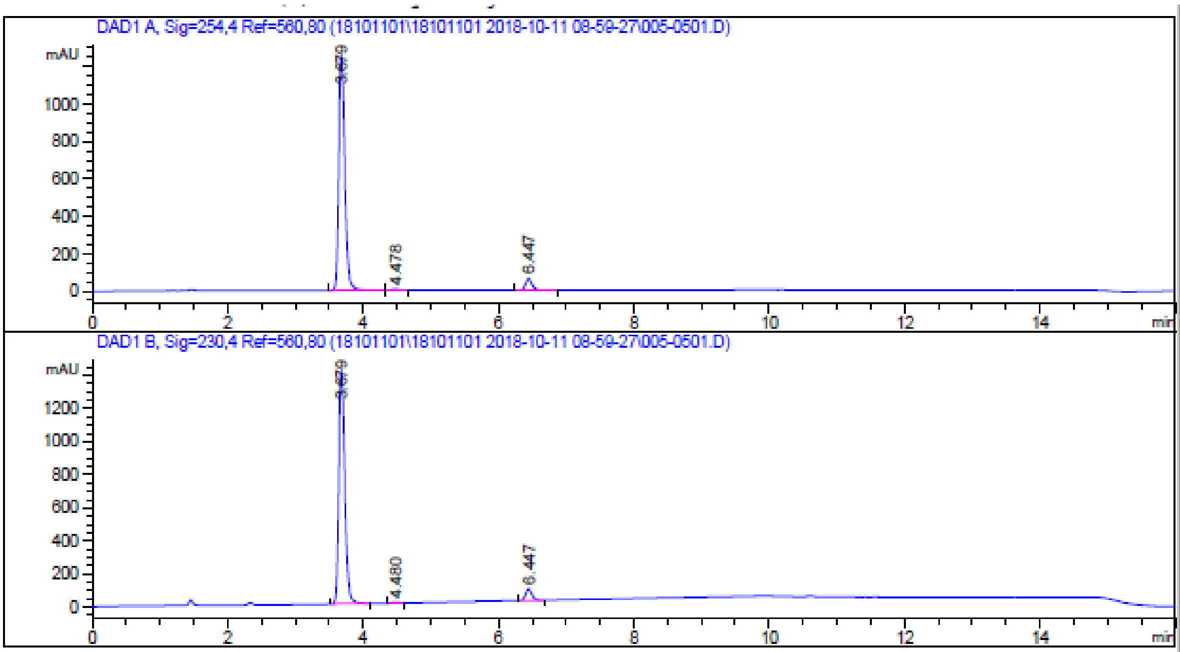

===

Area Percent Report

===

Sorted By : Signal
Multiplier: : 1.0000
Dilution: : 1.0000
Use Multiplier & Dilution Factor with ISTDs

Signal 1: DAD1 A, Sig=254,4 Ref=560,80

Peak #	RetTime [min]	Type	Width [min]	Area [mAU*s]	Height [mAU]	Area %
1	3.679	BB	0.0967	7791.60352	1250.34216	94.7345
2	4.478	BB	0.0918	34.85546	5.82729	0.4238
3	6.447	BB	0.0969	398.21310	62.05519	4.8417

*Figure S9. HPLC report of compound **10**.*

N-((2S,3R)-3-(benzyloxy)-1-((1-cyanocyclopropyl)amino)-1-oxobutan-2-yl)-3-(tert-butyl)-1-methyl-1H-pyrazole-5-carboxamide (**12**)

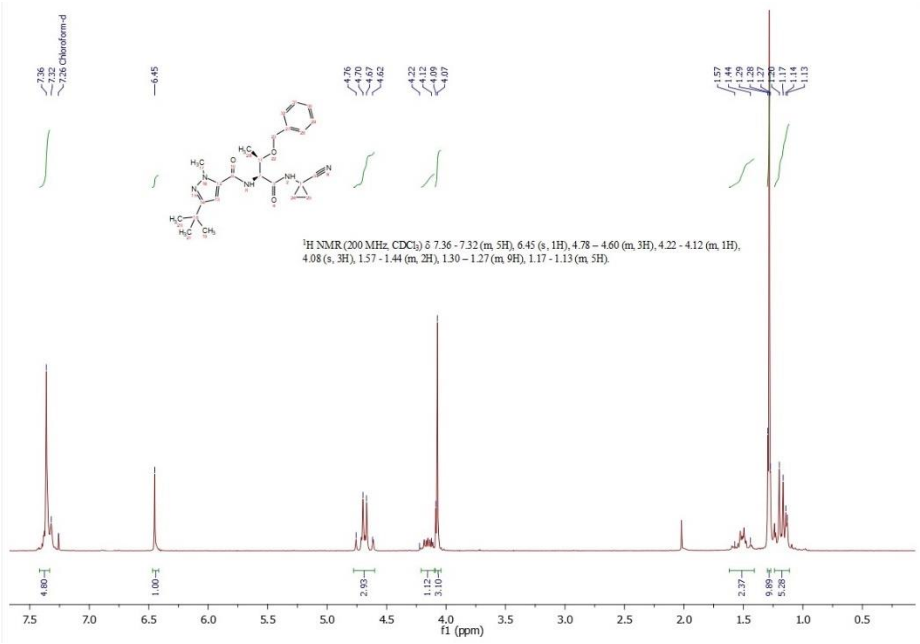

Figure S10. ^{1}H NMR (200 MHz, $CDCl_3$) of compound ***12****.*

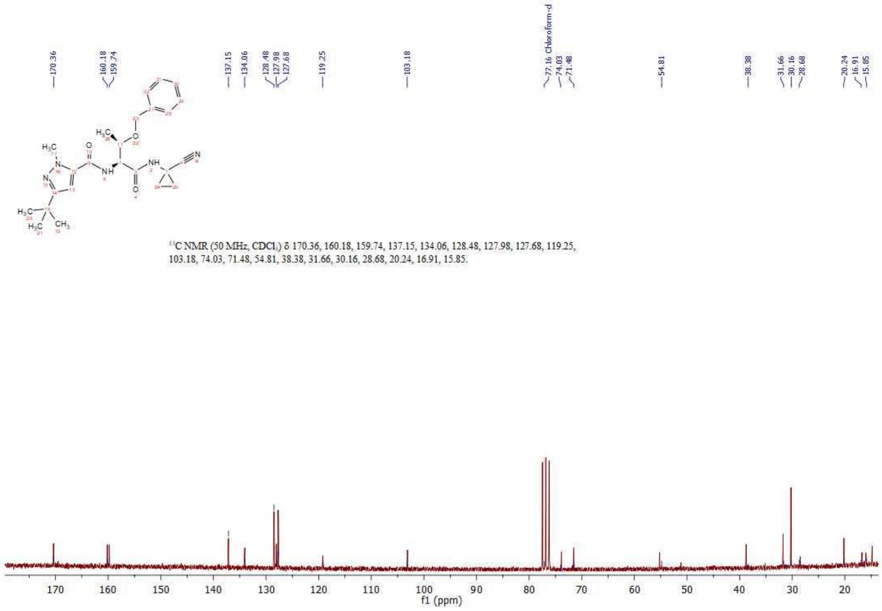

Figure S11 ^{13}C NMR (50 MHz, $CDCl_3$) of compound ***12****.*

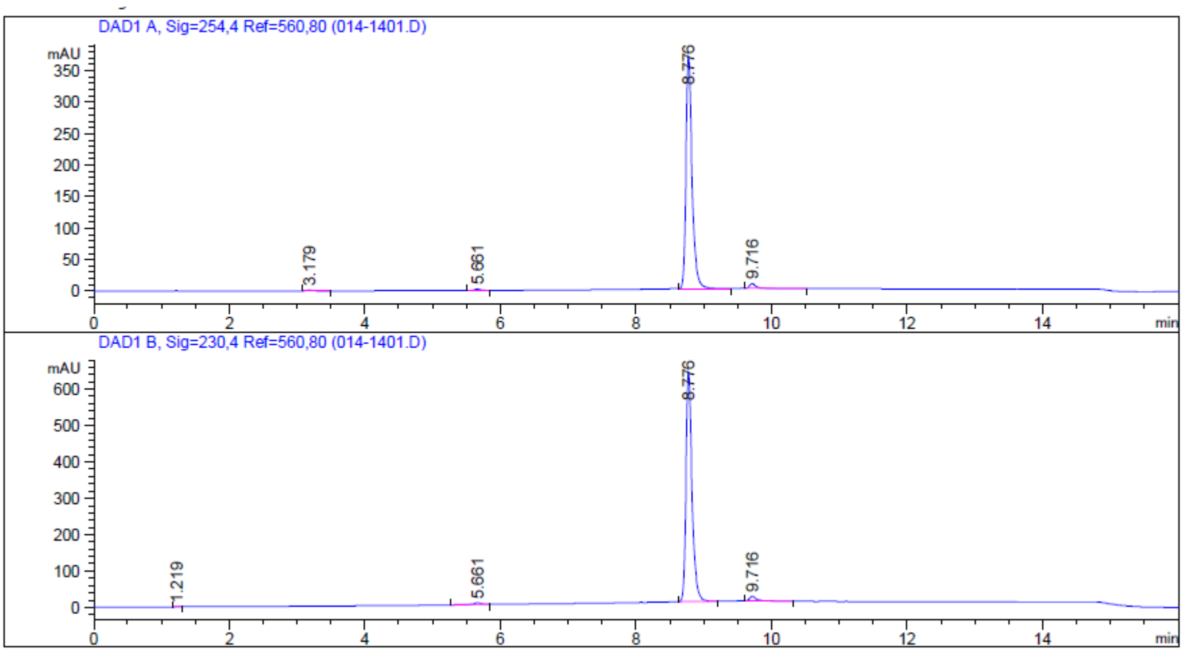

===

Area Percent Report

===

Sorted By : Signal
Multiplier: : 1.0000
Dilution: : 1.0000
Use Multiplier & Dilution Factor with ISTDs

Signal 1: DAD1 A, Sig=254,4 Ref=560,80

Peak #	RetTime [min]	Type	Width [min]	Area [mAU*s]	Height [mAU]	Area %
1	3.179	BB	0.0896	8.67321	1.45392	0.3687
2	5.661	BB	0.0991	13.63648	2.06427	0.5796
3	8.776	BB	0.0919	2282.44727	370.22195	97.0177
4	9.716	BB	0.0934	47.85194	7.60336	2.0340

*Figure S12. HPLC report of compound **12**.*

(S)-7-chloro-N-(1-((1-cyanocyclopropyl)amino)-1-oxo-3-phenylpropan-2-yl)quinoline-4-carboxamide (**13**)

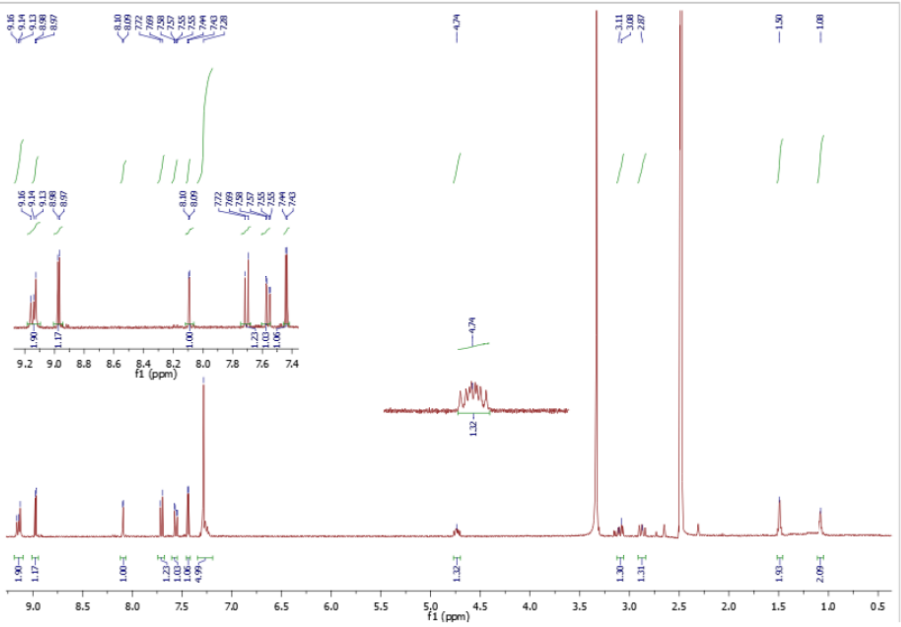

Figure S13. ^{1}*H-NMR (500 MHz, DMSO_d*$_6$*) of compound* ***13****.*

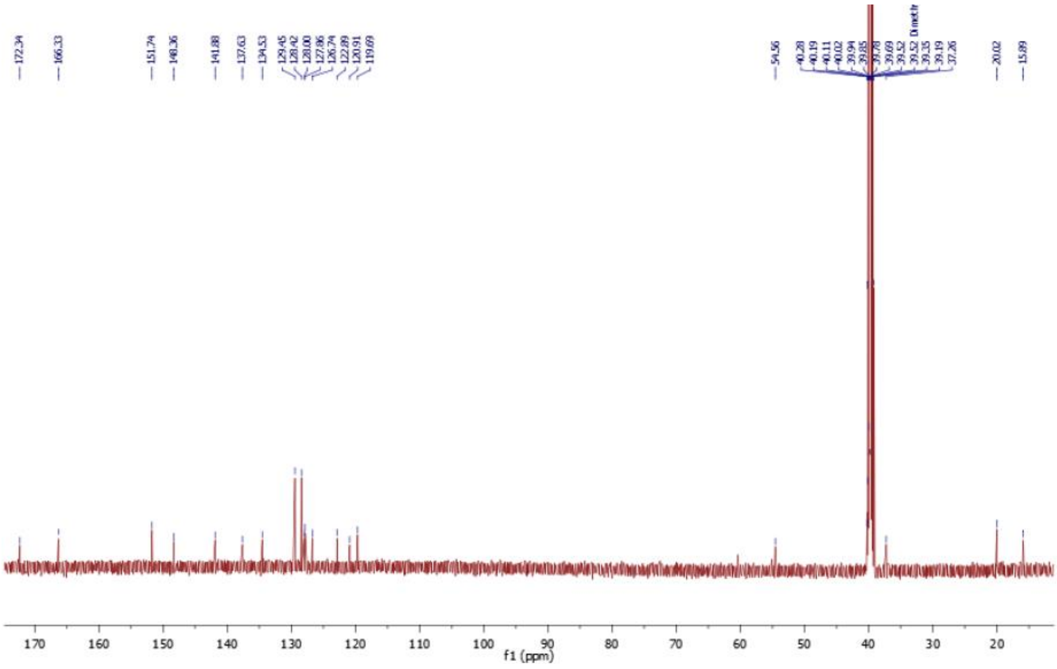

Figure S14. ^{13}*C NMR (125 MHz, DMSO_d*$_6$*) of compound* ***13****.*

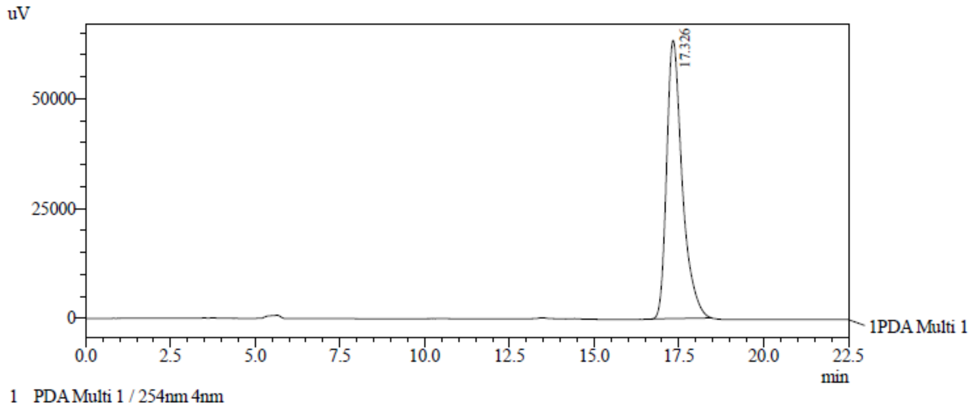

PeakTable

PDA Ch1 254nm 4nm

Peak#	Ret. Time	Area	Height	Area %	Height %
1	17.326	2032223	63401	100.000	100.000
Total		2032223	63401	100.000	100.000

Figure S15. HPLC report for compound ***13****.*

*(S)-7-chloro-N-(1-((1-cyanocyclopropyl)amino)-4-methyl-1-oxopentan-2-yl)quinoline-4-carboxamide (**14**)*

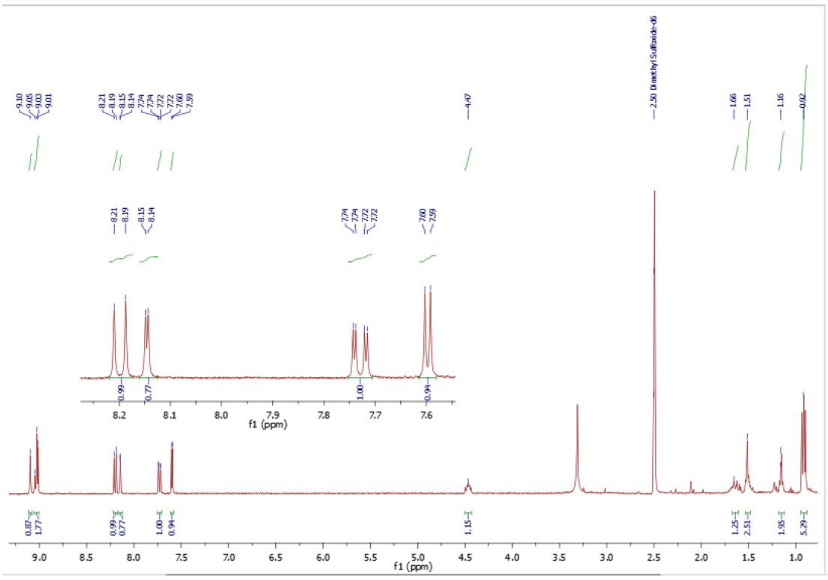

*Figure S16. ^{1}H-NMR (400 MHz, DMSO_d$_{6}$) of compound **14**.*

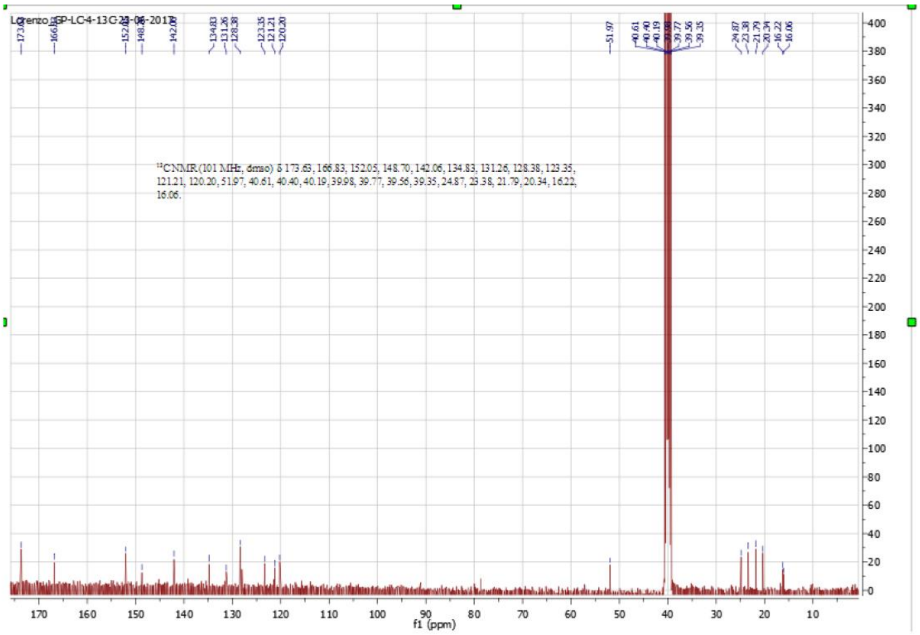

*Figure S17. ^{13}C NMR (100 MHz, DMSO_d$_{6}$) of compound **14**.*

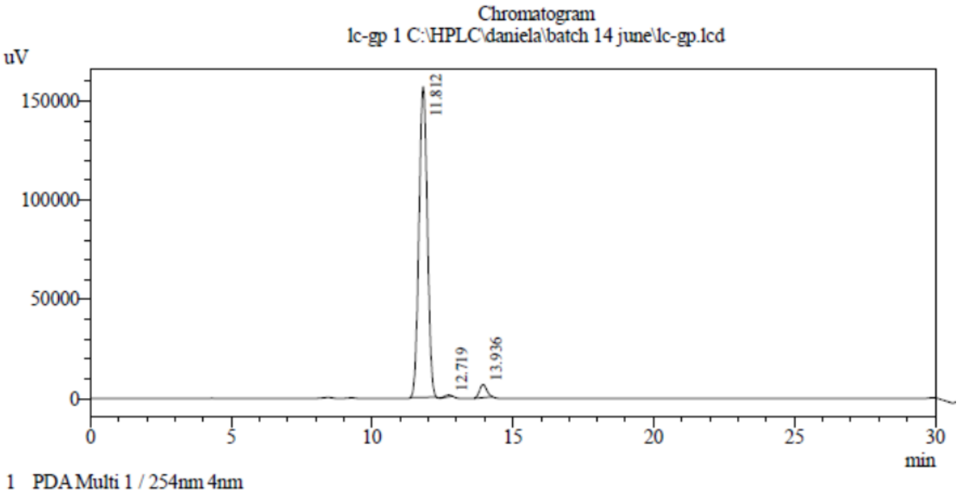

PeakTable

PDA Ch1 254nm 4nm

Peak#	Ret. Time	Area	Height	Area %	Height %
1	11.812	3230825	156430	96.220	95.452
2	12.719	12767	881	0.380	0.537
3	13.936	114159	6573	3.400	4.011
Total		3357751	163884	100.000	100.000

Figure S18. HPLC report ofr compound ***14****.*

*(S)-7-chloro-N-(1-((1-cyanocyclopropyl)amino)-3-(1H-indol-3-yl)-1-oxopropan-2-yl)quinoline-4-carboxamide (**15**)*

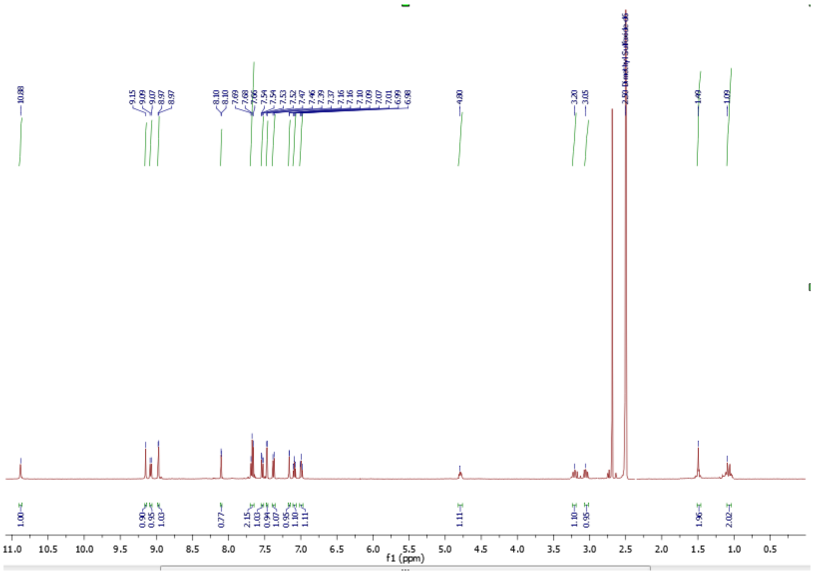

*Figure S19. ^{1}H-NMR (500MHz, DMSO_d$_{6}$) for compound **15**.*

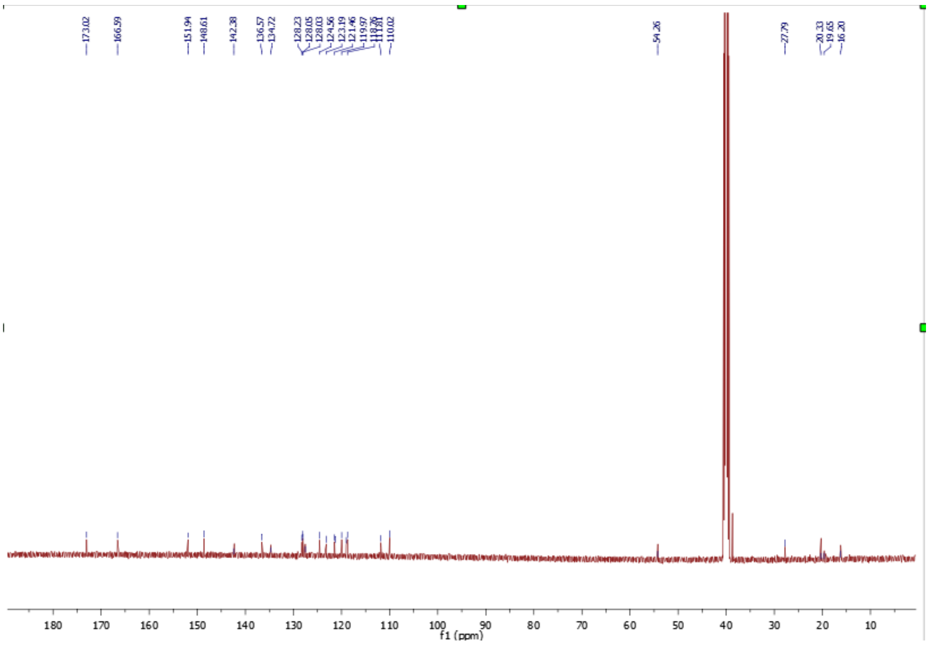

*Figure S20. ^{13}C NMR (125 MHz, DMSO_d$_{6}$) for compound **15**.*

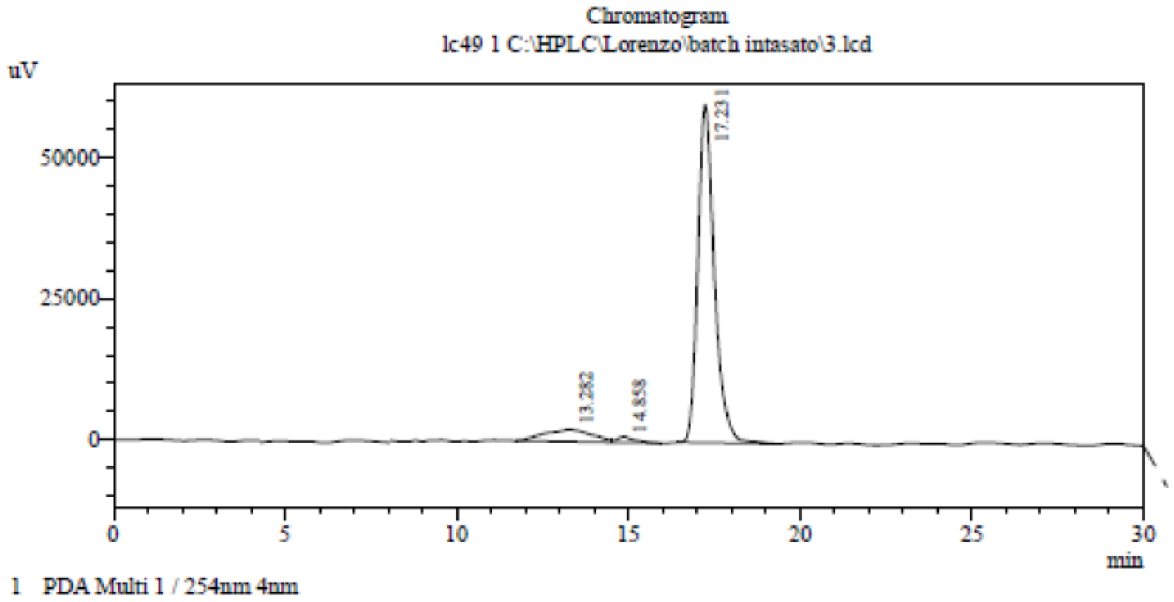

PeakTable

PDA Ch1 254nm 4nm

Peak#	Ret. Time	Area	Height	Area %	Height %
1	13.282	201433	2122	8.939	3.363
2	14.858	36316	1016	1.612	1.610
3	17.231	2015580	59950	89.449	95.026
Total		2253328	63088	100.000	100.000

Figure S21. HPLC report for compound ***15****.*

*(S)-3-((1-((1-cyanocyclopropyl)amino)-1-oxo-3-phenylpropan-2-yl)carbamoyl)phenyl benzoate (**16**)*

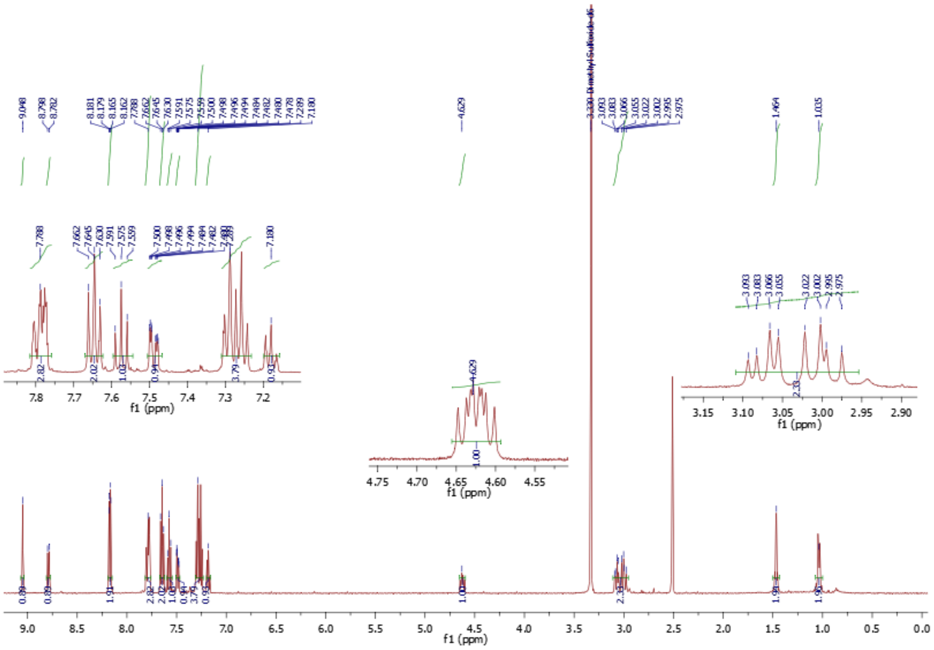

*Figure S22. ^{1}H-NMR (500MHz, DMSO_d$_6$) for compound **16**.*

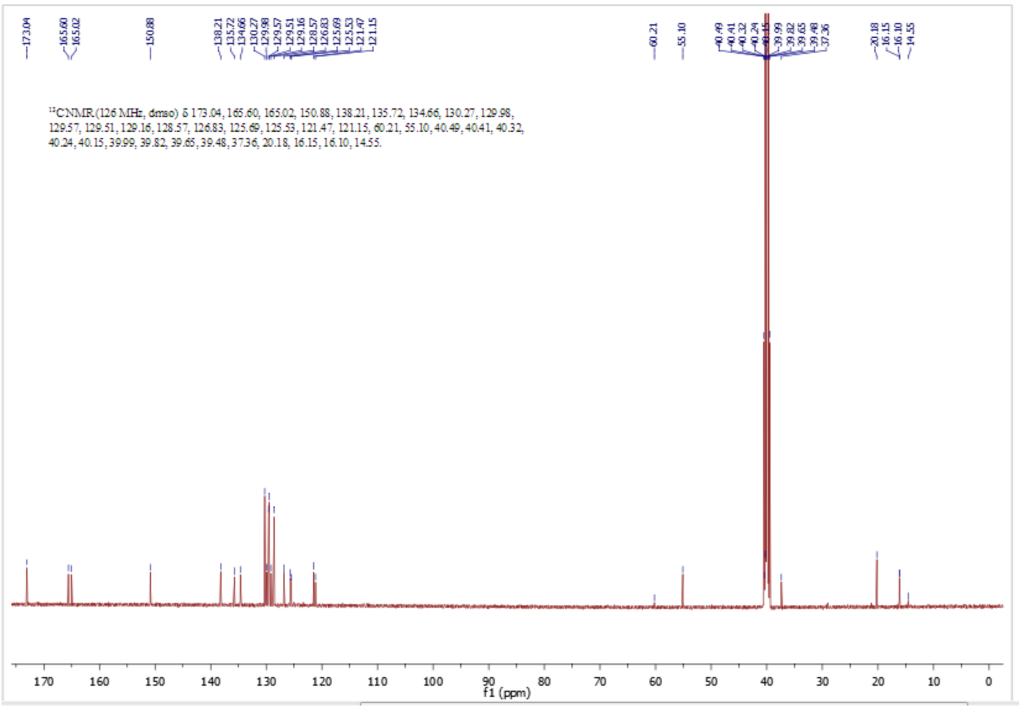

*Figure S23. ^{13}C NMR (125 MHz, DMSO_d$_6$) for compound **16**.*

Acquisition Parameter

Ion Source Type	ESI	Ion Polarity	Positive	Alternating Ion Polarity	off
Mass Range Mode	Enhanced Resolution	Scan Begin	100 m/z	Scan End	1200 m/z
Accumulation Time	488 µs	RF Level	67 %	Trap Drive	49.8
SPS Target Mass	450 m/z	Averages	5 Spectra	n/a	n/a

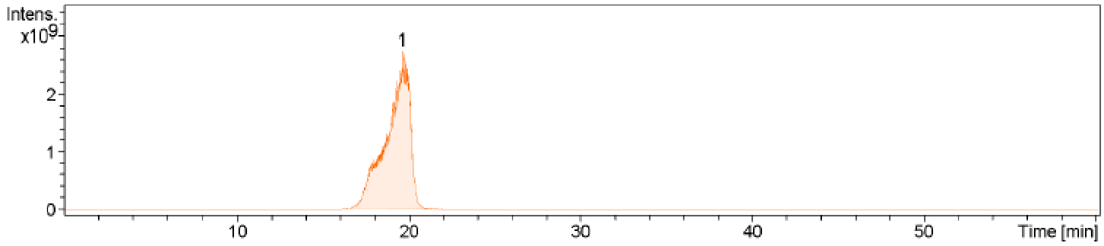

#	RT [min]	Chromatogram	Area	Area %	S/N
1	19.6	EIC C27H23N3O4 [M+H]+ 454.18 All MS	239942025216	100.00	2873.6

Cmpd 1, 19.6 min

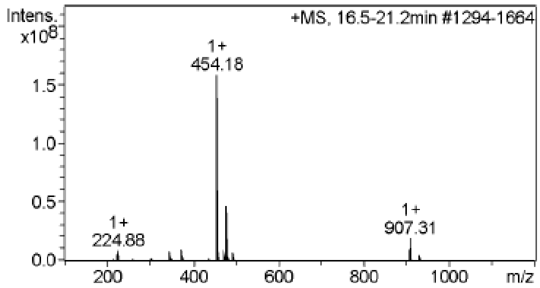

Figure S24. HPLC report for compound ***16***.

*(S)-6-amino-N-(1-((1-cyanocyclopropyl)amino)-4-methyl-1-oxopentan-2-yl)nicotinamide (**17**)*

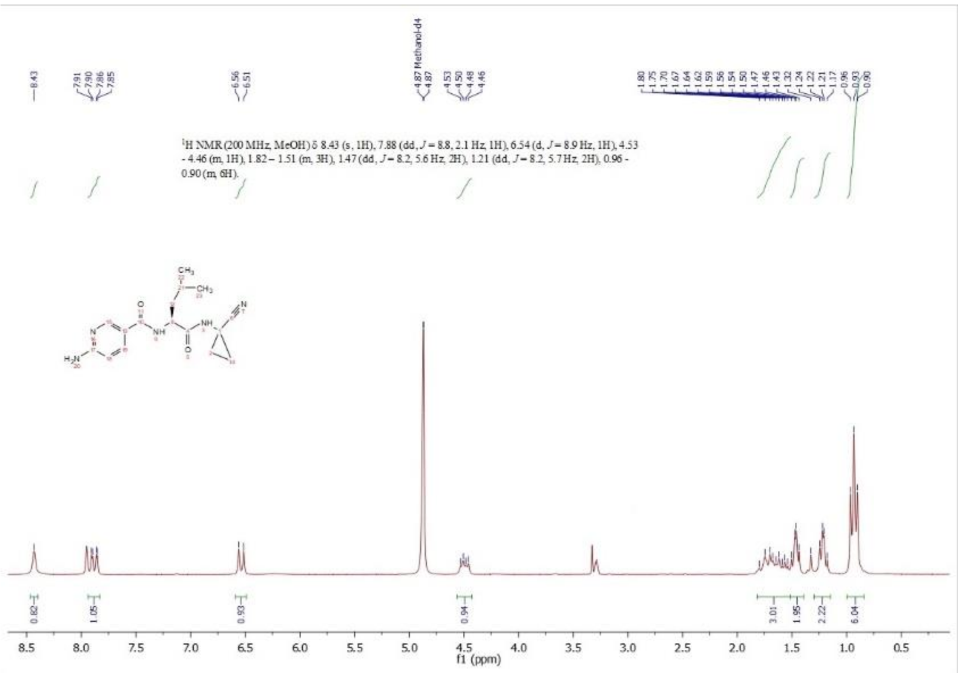

*Figure S25. ^{1}H-NMR (200MHz, CD_3OD) for compound **17**.*

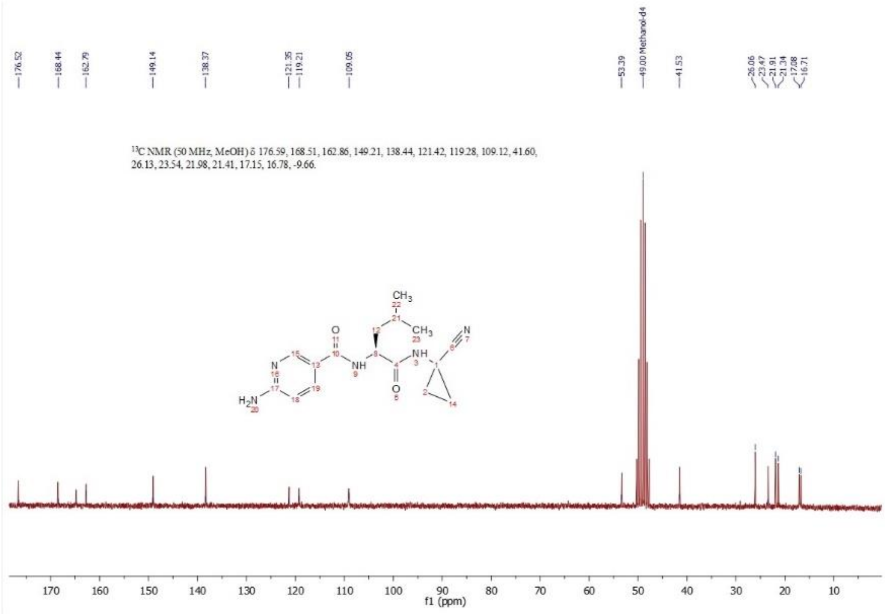

*Figure S26. ^{13}C NMR (50 MHz, CD_3OD) for compound **17**.*

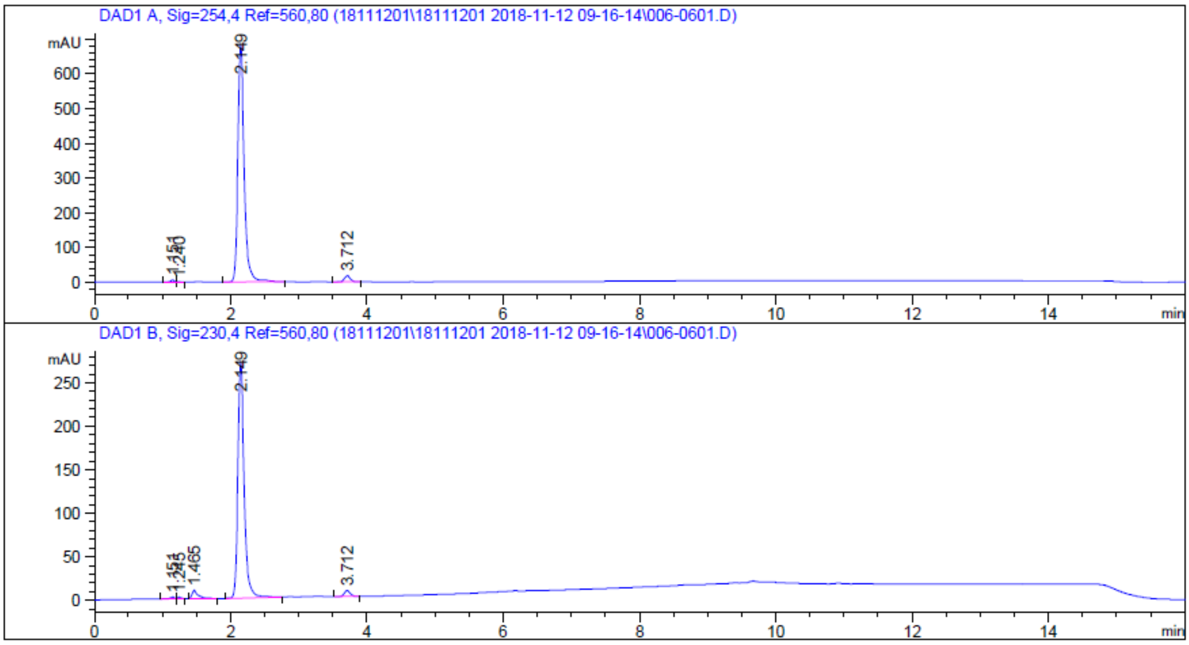

===

Area Percent Report

===

Sorted By : Signal
Multiplier: : 1.0000
Dilution: : 1.0000
Use Multiplier & Dilution Factor with ISTDs

Signal 1: DAD1 A, Sig=254,4 Ref=560,80

Peak #	RetTime [min]	Type	Width [min]	Area [mAU*s]	Height [mAU]	Area %
1	1.151	BV	0.0615	22.94661	5.64046	0.5087
2	1.240	VB	0.0474	4.44436	1.46560	0.0985
3	2.149	BB	0.0968	4379.34082	682.89142	97.0893
4	3.712	BB	0.0916	103.89816	17.41061	2.3034

*Figure S27. HPLC report for compound **17**.*

*(S)-N-(1-((1-cyanocyclopropyl)amino)-4-methyl-1-oxopentan-2-yl)-1H-pyrrolo[2,3-b]pyridine-5-carboxamide (**18**)*

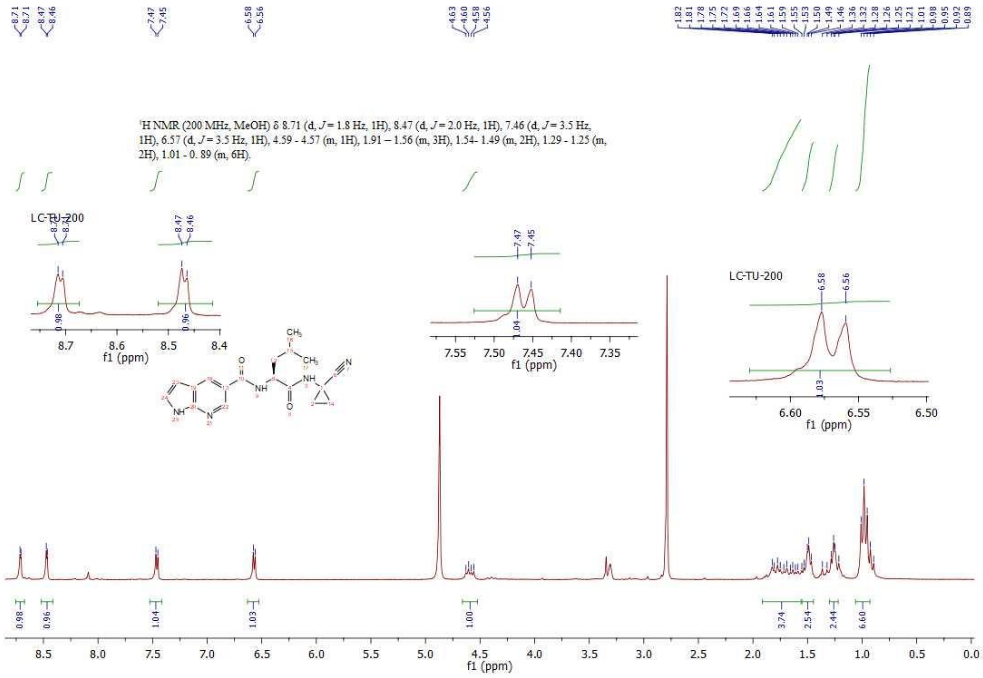

*Figure S28. ^{1}H-NMR (200MHz, CD_3OD) of compound **18**.*

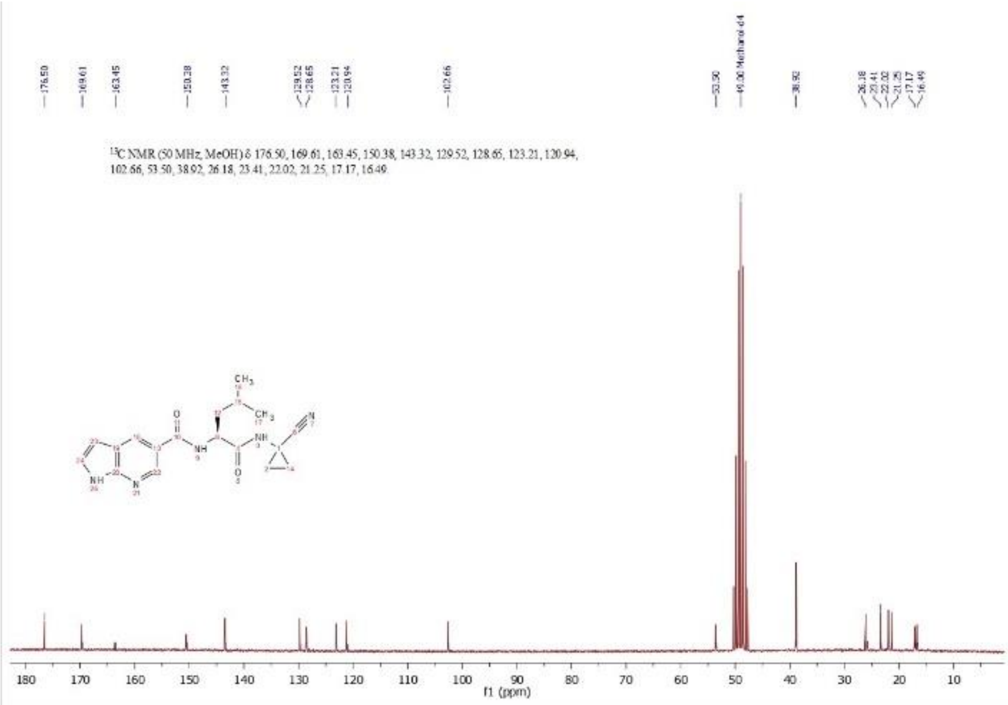

*Figure S29. ^{13}C NMR (50 MHz, CD_3OD) of compound **18**.*

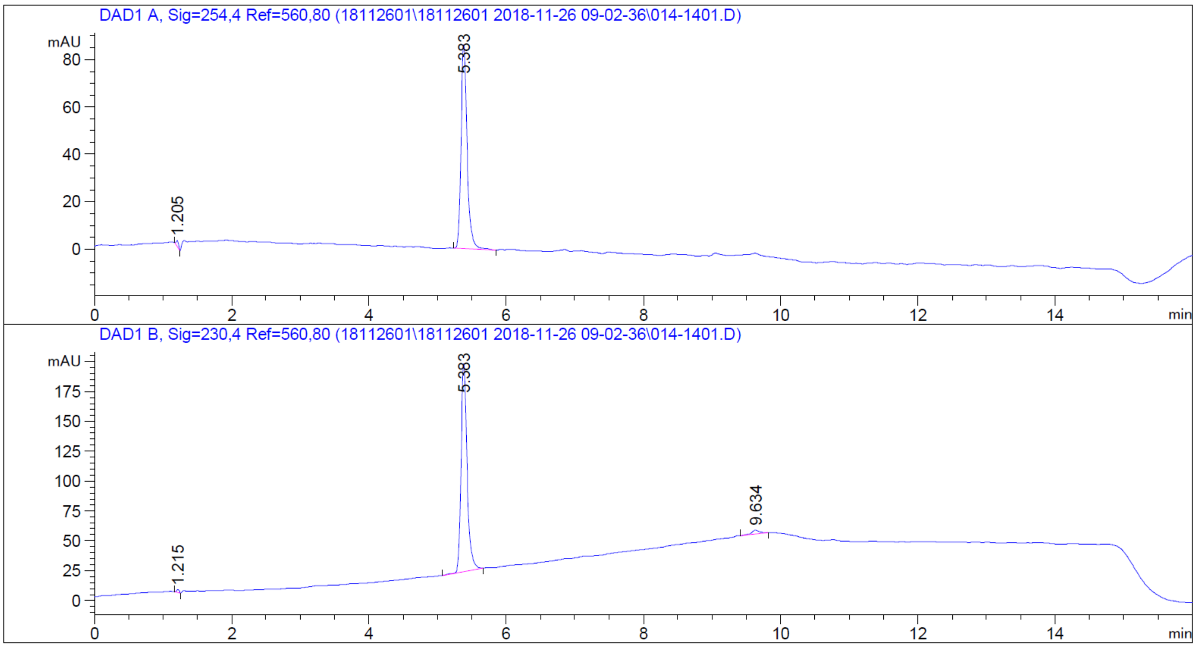


```
=====================================================================
                         Area Percent Report
=====================================================================

Sorted By             :      Signal
Multiplier:                   :       1.0000
Dilution:                     :       1.0000
Use Multiplier & Dilution Factor with ISTDs


Signal 1: DAD1 A, Sig=254,4 Ref=560,80

Peak RetTime Type  Width     Area      Height     Area
  #   [min]        [min]   [mAU*s]     [mAU]       %
----|-------|----|-------|----------|----------|--------|
   1   1.205 BB    0.0369    5.31597    2.30899   0.9967
   2   5.383 BB    0.0935  528.03094   86.14277  99.0033

Totals :                   533.34692   88.45176
```

*Figure S30. HPLC report for compound **18**.*

*3-(tert-butyl)-N-((2S,3R)-1-((1-cyanocyclopropyl)amino)-3-hydroxy-1-oxobutan-2-yl)-1-methyl-1H-pyrazole-5-carboxamide (**19**)*

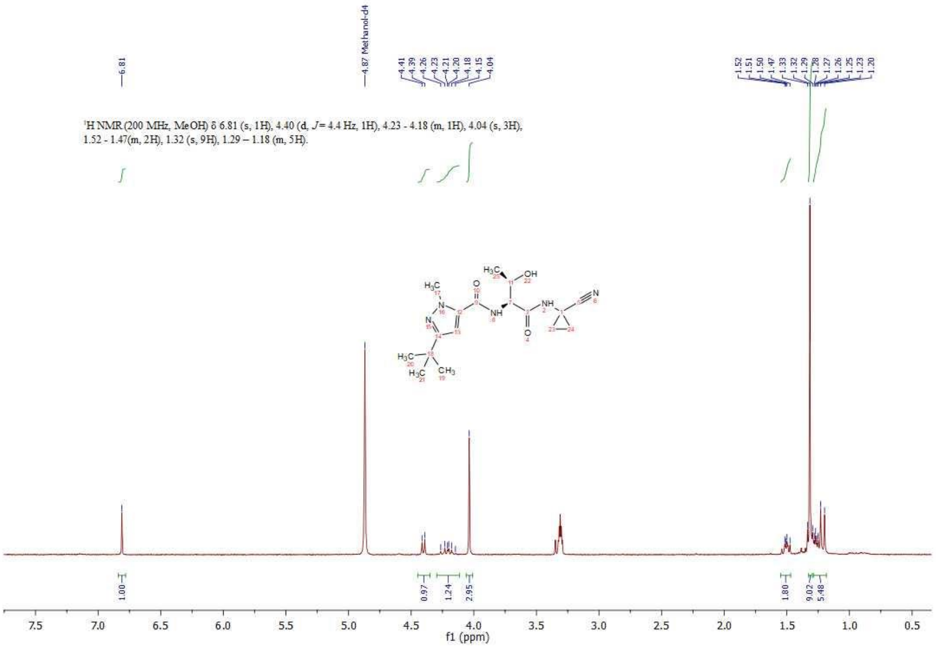

*Figure S31. ^{1}H-NMR (200 MHz, CD_3OD) of compound **19**.*

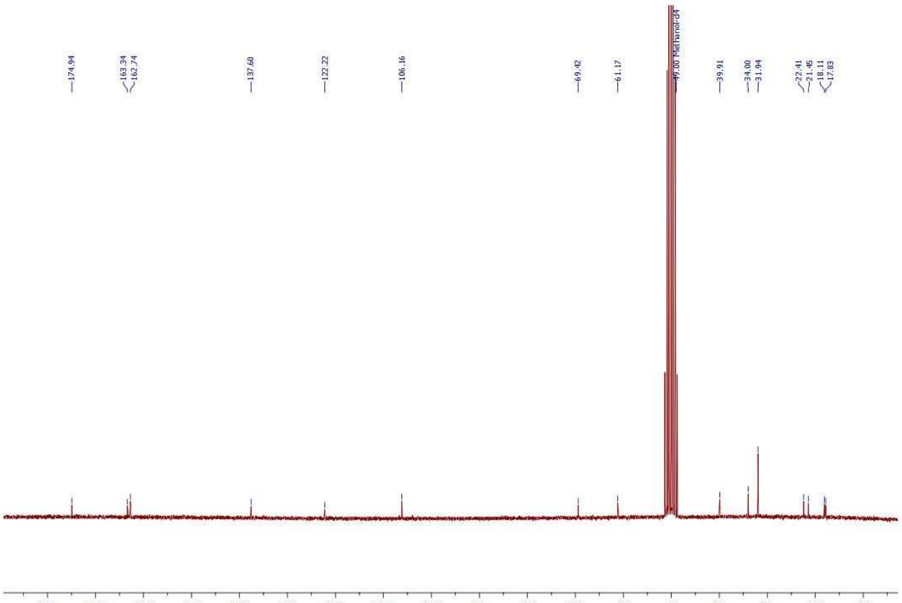

*Figure S 32. ^{13}C NMR (50 MHz, CD_3OD) of compound **19**.*

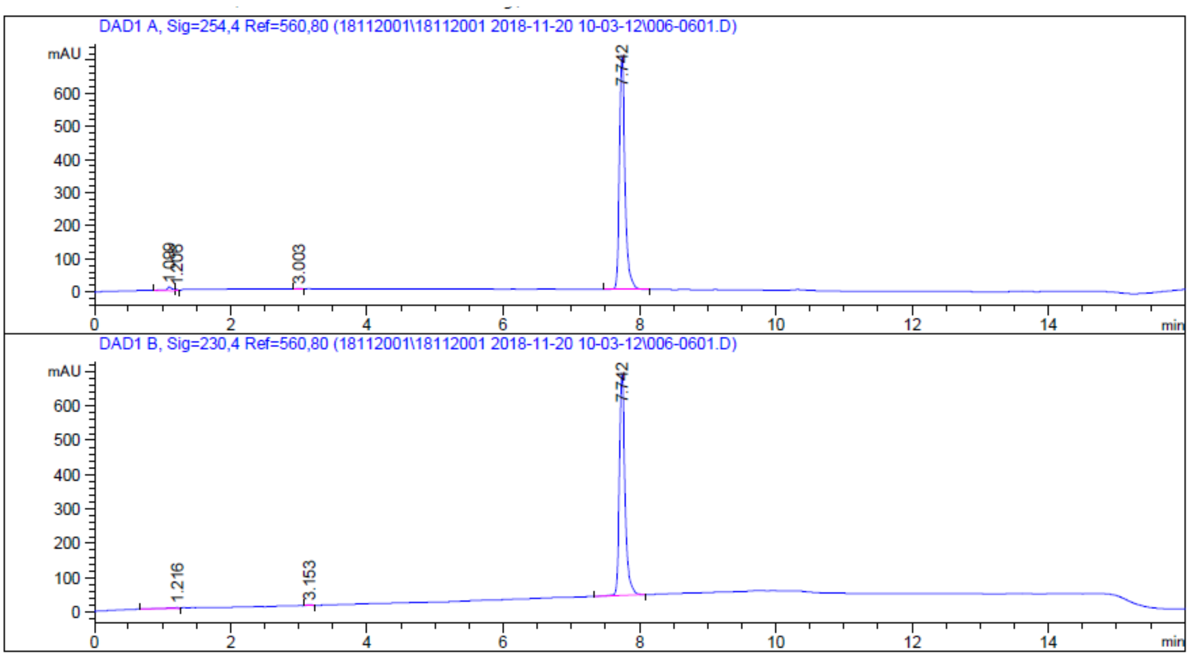

===

Area Percent Report

===

Sorted By : Signal
Multiplier: : 1.0000
Dilution: : 1.0000
Use Multiplier & Dilution Factor with ISTDs

Signal 1: DAD1 A, Sig=254,4 Ref=560,80

Peak #	RetTime [min]	Type	Width [min]	Area [mAU*s]	Height [mAU]	Area %
1	1.099	BV	0.0779	62.71178	11.45192	1.5285
2	1.206	VB	0.0395	10.38106	4.11194	0.2530
3	3.003	BB	0.0730	7.52391	1.65465	0.1834
4	7.742	BB	0.0866	4022.22974	704.58551	98.0351

*Figure S33. HPLC report for compound **19***

*3-(tert-butyl)-N-((S)-1-(((S)-1-cyano-2-phenylethyl)amino)-1-oxo-3-phenylpropan-2-yl)-1-methyl-1H-pyrazole-5-carboxamide (**50**)*

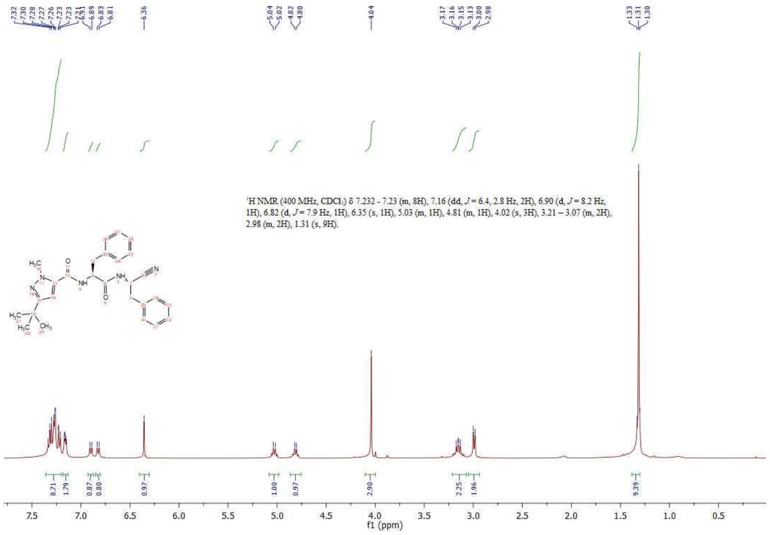

*Figure S34. ^{1}H-NMR (400 MHz, $CDCl_3$) of compound **50**.*

*Figure S35. ^{13}C NMR (100 MHz, $CDCl_3$) of compound **50**.*

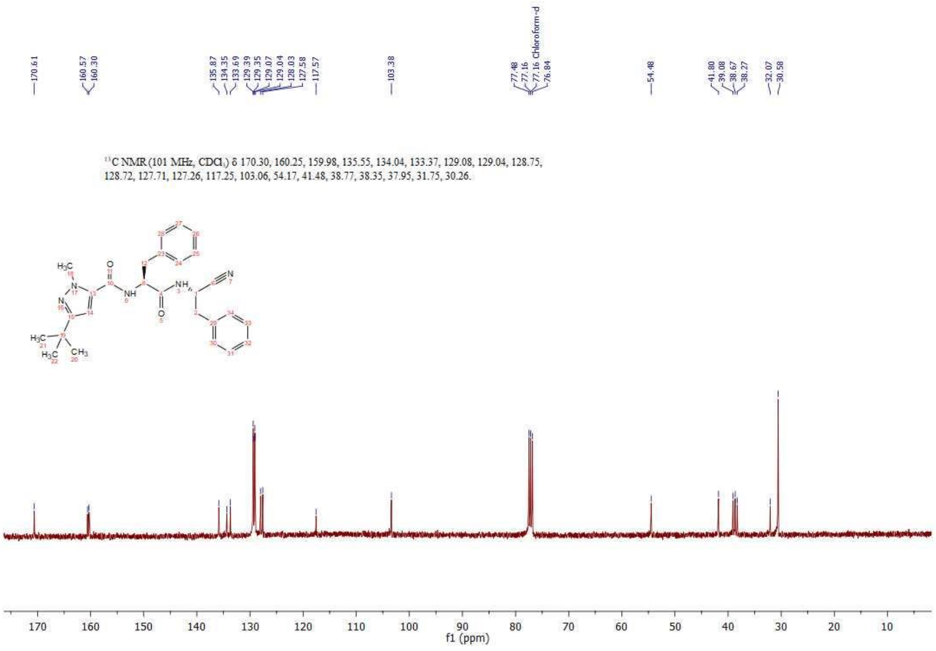

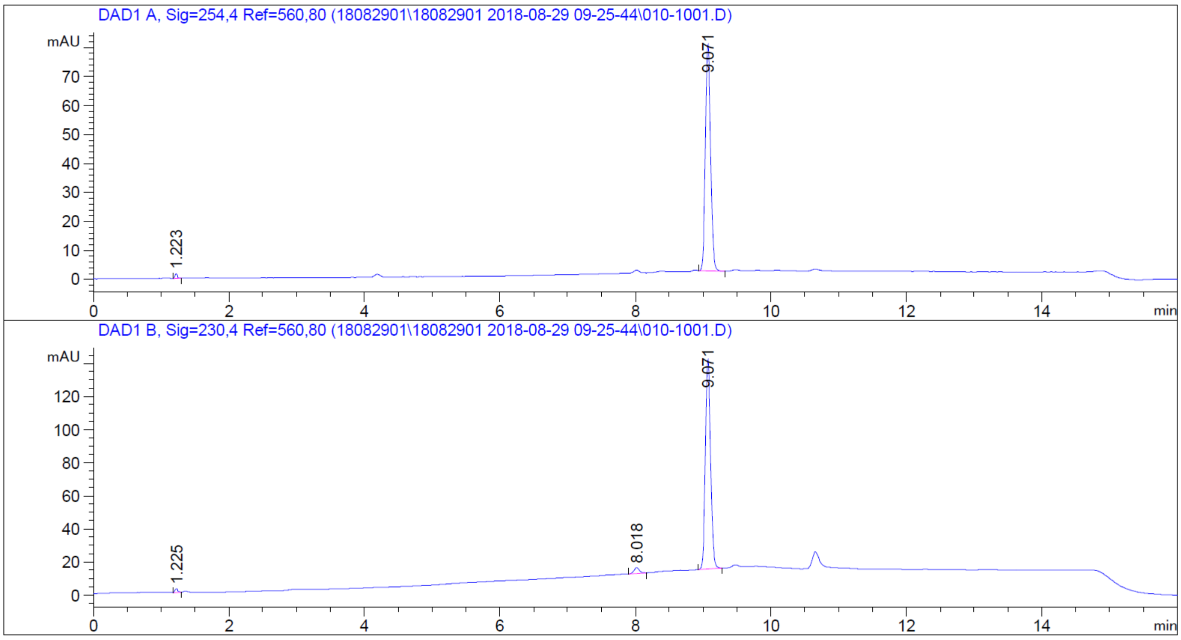

===

Area Percent Report

===

Sorted By : Signal
Multiplier: : 1.0000
Dilution: : 1.0000
Use Multiplier & Dilution Factor with ISTDs

Signal 1: DAD1 A, Sig=254,4 Ref=560,80

Peak #	RetTime [min]	Type	Width [min]	Area [mAU*s]	Height [mAU]	Area %
1	1.223	BB	0.0418	4.10219	1.60595	0.9748
2	9.071	BB	0.0821	416.71085	78.30849	99.0252

Totals : 420.81304 79.91444

*Figure S36. HPLC report of compound **50**.*

*3-(tert-butyl)-N-((S)-1-(((R)-1-cyano-2-phenylethyl)amino)-1-oxo-3-phenylpropan-2-yl)-1-methyl-1H-pyrazole-5-carboxamide (**51**)*

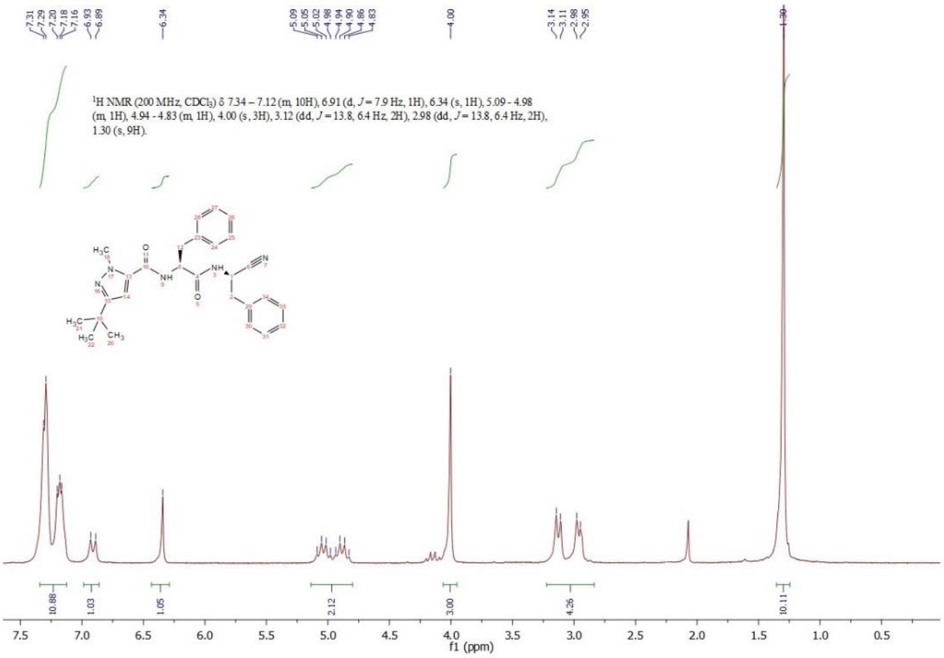

Figure S37. ^{1}*H-NMR (200 MHz,* $CDCl_3$*) of compound* ***51****.*

*Figure S38. ^{13}C NMR (50 MHz, $CDCl_3$) for compound **51**.*

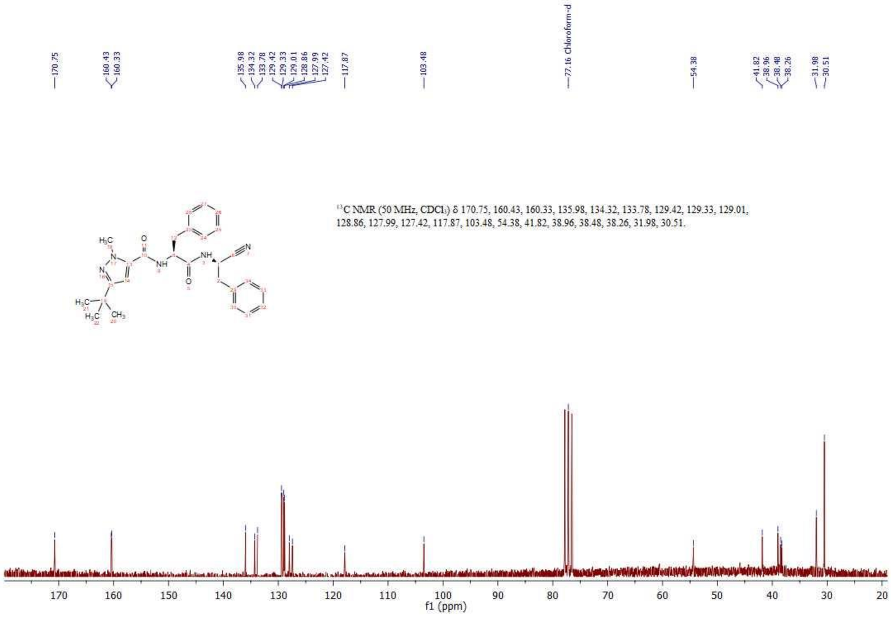

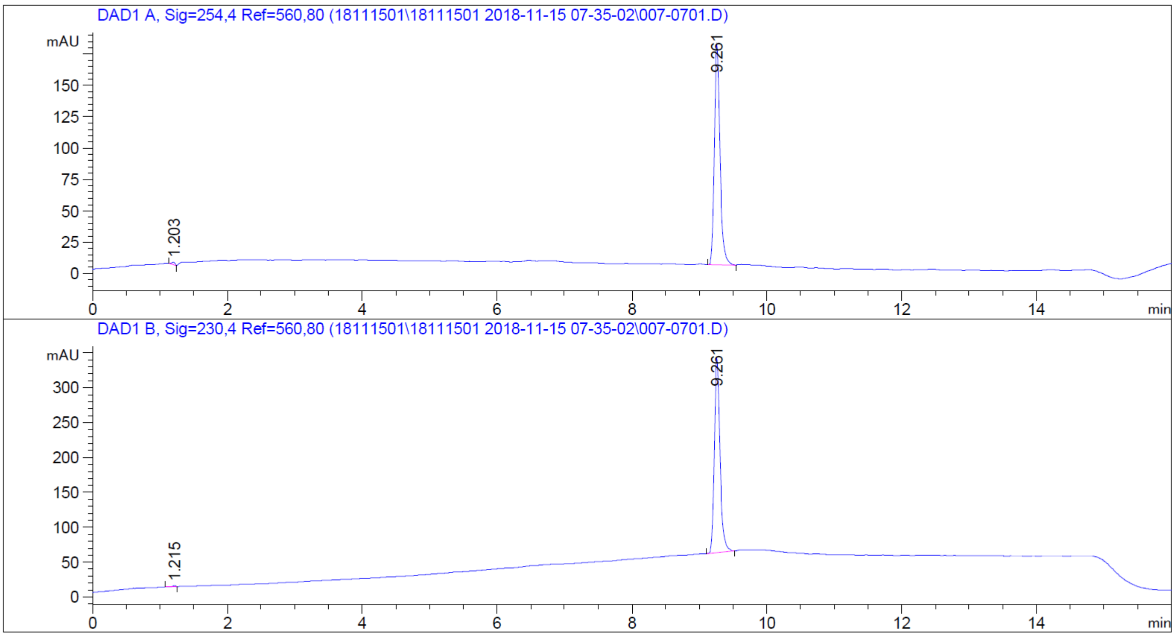

===

Area Percent Report

===

Sorted By : Signal
Multiplier: : 1.0000
Dilution: : 1.0000
Use Multiplier & Dilution Factor with ISTDs

Signal 1: DAD1 A, Sig=254,4 Ref=560,80

Peak #	RetTime [min]	Type	Width [min]	Area [mAU*s]	Height [mAU]	Area %
1	1.203	BB	0.0488	6.71654	2.12781	0.6557
2	9.261	BB	0.0896	1017.58386	175.51555	99.3443

Totals : 1024.30040 177.64336

Figure S39. HPLC report of compound ***51.***

*3-(tert-butyl)-N-((S)-1-(((S)-1-cyano-3-methylbutyl)amino)-1-oxo-3-phenylpropan-2-yl)-1-methyl-1H-pyrazole-5-carboxamide (**52**)*

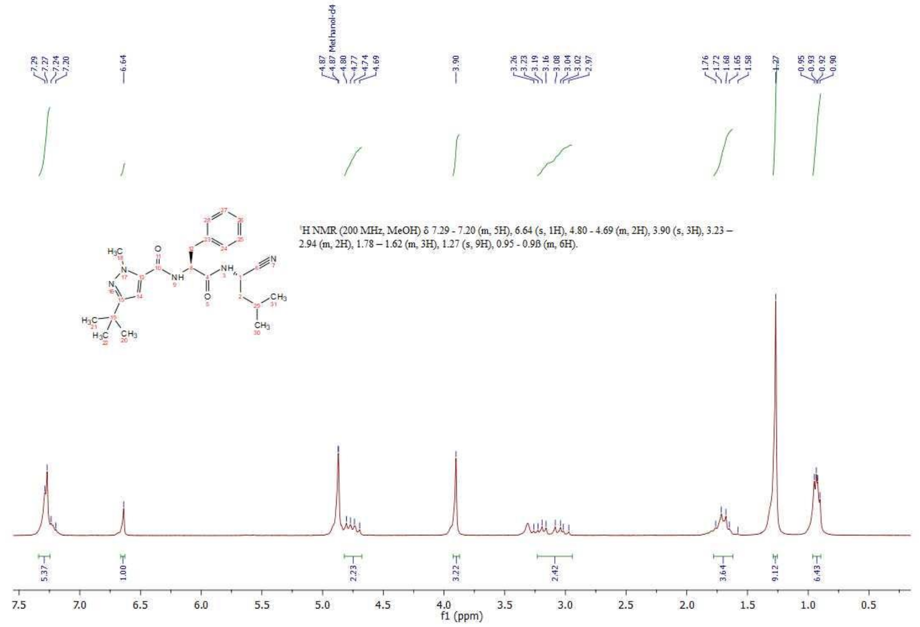

*Figure S40. ^{1}H-NMR (200 MHz, CD_3OD) of compound **52**.*

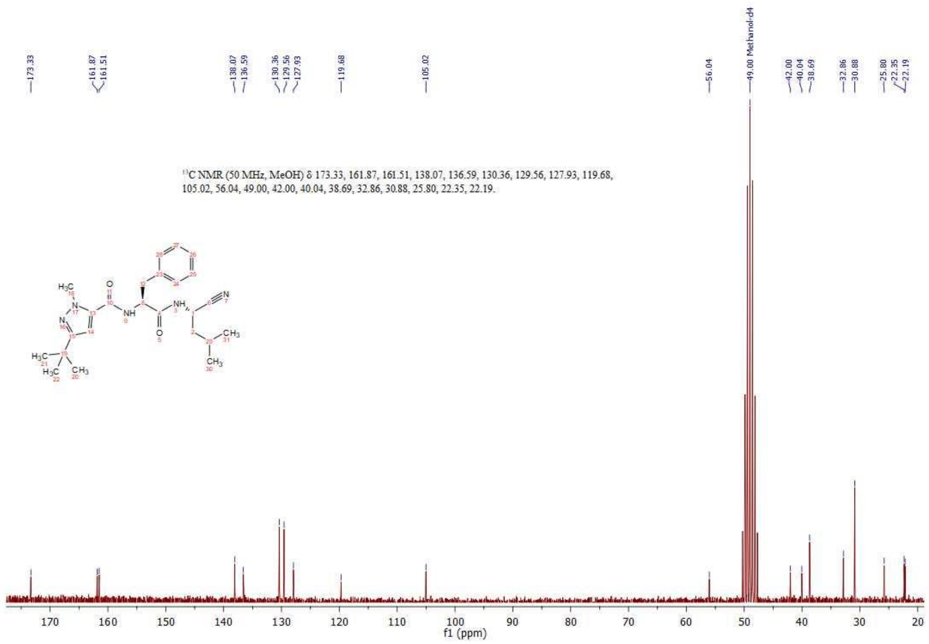

*Figure S41. ^{13}C NMR (50 MHz, CD_3OD) of compound **52**.*

Additional Info : Peak(s) manually integrated

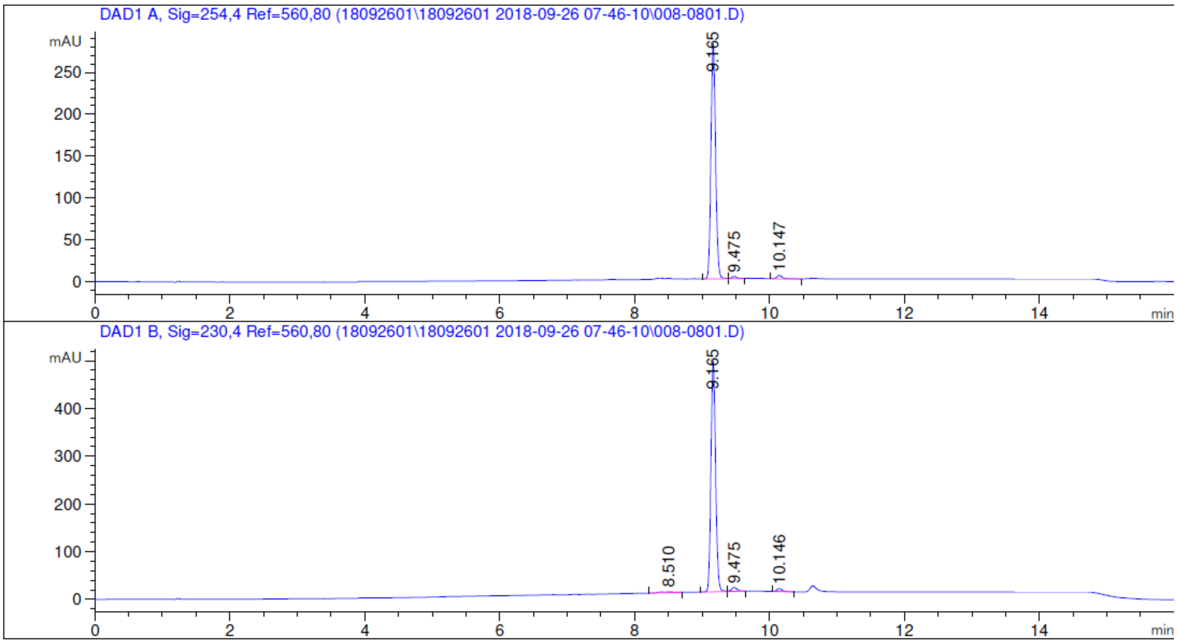

===

Area Percent Report

===

Sorted By : Signal
Multiplier: : 1.0000
Dilution: : 1.0000
Use Multiplier & Dilution Factor with ISTDs

Signal 1: DAD1 A, Sig=254,4 Ref=560,80

Peak #	RetTime [min]	Type	Width [min]	Area [mAU*s]	Height [mAU]	Area %
1	9.165	BV	0.0733	1334.22681	281.32031	97.4268
2	9.475	VB	0.0826	14.42373	2.60572	1.0532
3	10.147	BB	0.0817	20.81604	3.81424	1.5200

Totals : 1369.46658 287.74027

*Figure S42. HPLC report of compound **52**.*

*3-(tert-butyl)-N-((S)-1-(((S)-1-cyano-3-methylbutyl)amino)-1-oxo-3-phenylpropan-2-yl)-1-methyl-1H-pyrazole-5-carboxamide (**53**)*

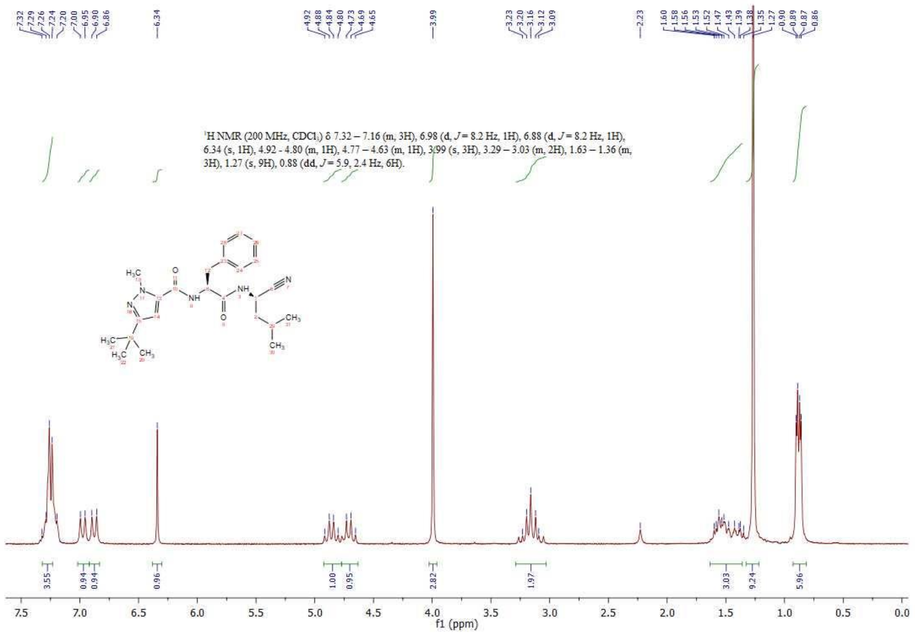

*Figure S43. ^{1}H-NMR (200 MHz, $CDCl_3$) of compound **53**.*

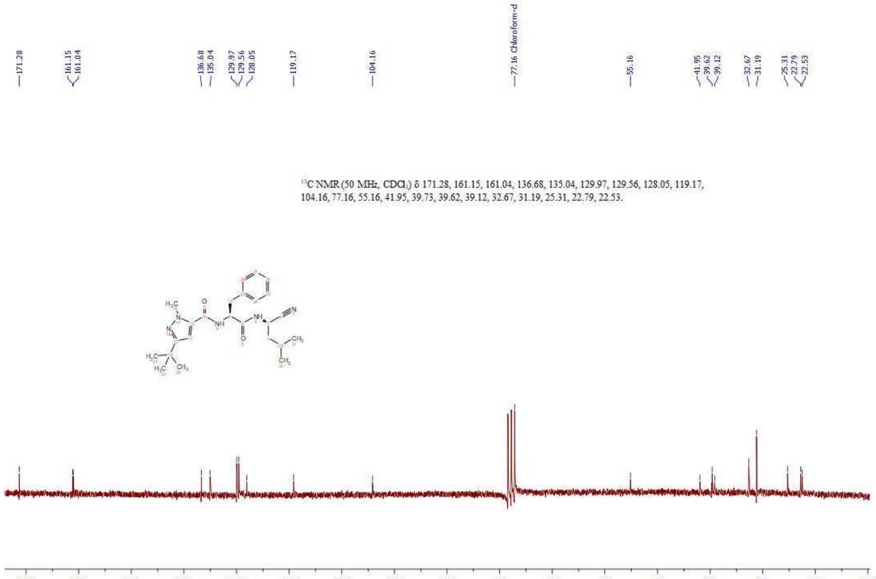

*Figure S44. ^{13}C NMR (50 MHz, $CDCl_3$) of compound **53**.*

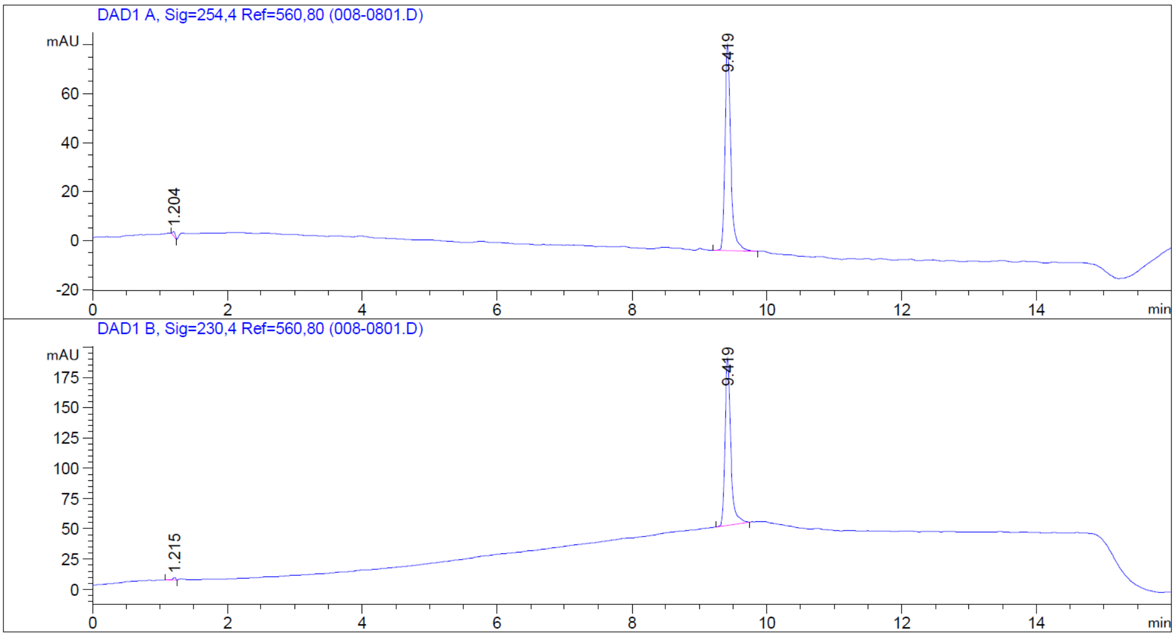

===

Area Percent Report

===

Sorted By : Signal
Multiplier: : 1.0000
Dilution: : 1.0000
Use Multiplier & Dilution Factor with ISTDs

Signal 1: DAD1 A, Sig=254,4 Ref=560,80

Peak #	RetTime [min]	Type	Width [min]	Area [mAU*s]	Height [mAU]	Area %
1	1.204	BB	0.0380	4.21333	1.75584	0.8310
2	9.419	BB	0.0895	502.81897	84.36648	99.1690

Totals : 507.03230 86.12231

*Figure S45. HPLC report of compound **53**.*

*3-(tert-butyl)-N-((S)-1-(((S)-2-(3-chlorophenyl)-1-cyanoethyl)amino)-1-oxo-3-phenylpropan-2-yl)-1-methyl-1H-pyrazole-5-carboxamide (**54**)*

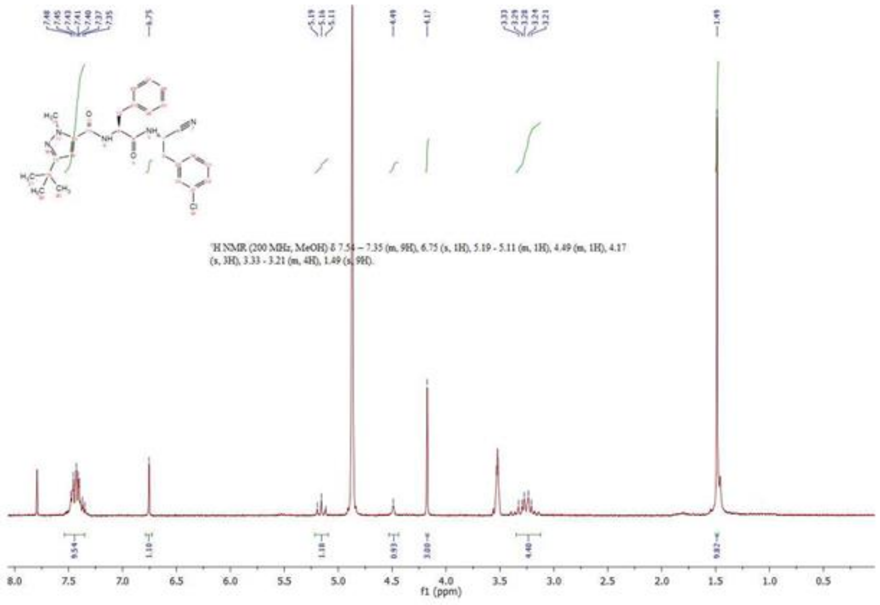

*Figure S46. ^{1}H-NMR (200 MHz, CD_3OD) of compound **54**.*

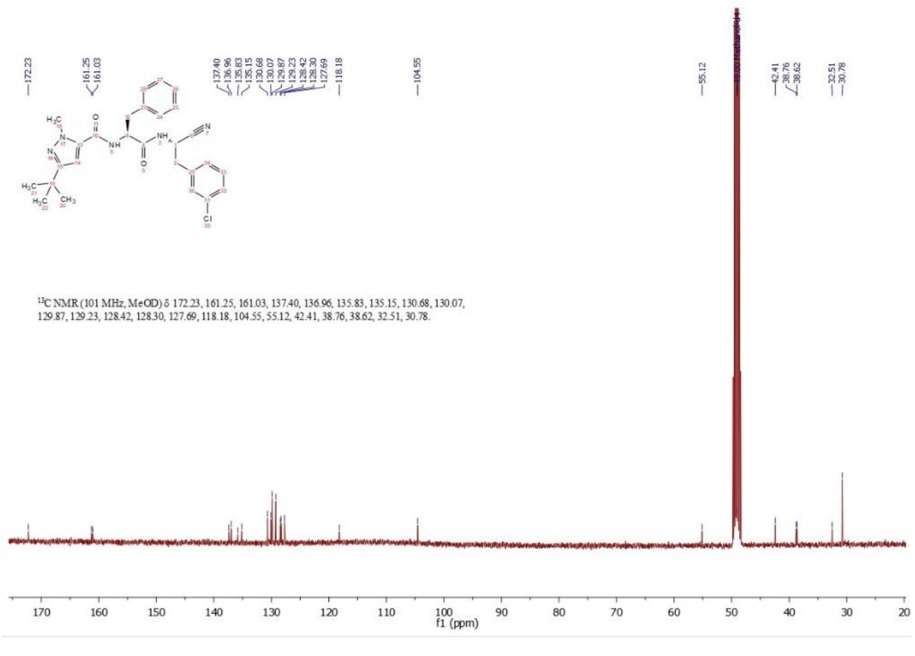

*Figure S47. ^{13}C NMR (50 MHz, CD_3OD) of compound **54**.*

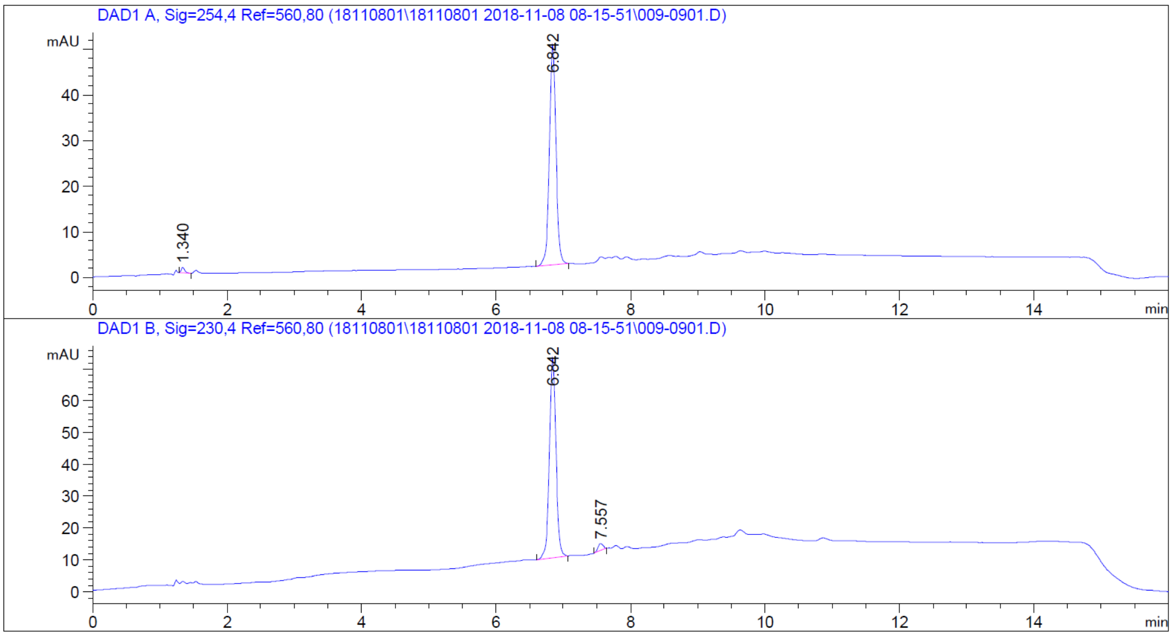

===

Area Percent Report

===

Sorted By : Signal
Multiplier: : 1.0000
Dilution: : 1.0000
Use Multiplier & Dilution Factor with ISTDs

Signal 1: DAD1 A, Sig=254,4 Ref=560,80

Peak #	RetTime [min]	Type	Width [min]	Area [mAU*s]	Height [mAU]	Area %
1	1.340	BB	0.0572	4.46968	1.20623	1.3366
2	6.842	BB	0.1057	329.93869	48.26745	98.6634

Totals : 334.40837 49.47368

*Figure S48. HPLC report of compound **54***

*3-(tert-butyl)-N-((S)-1-(((S)-1-cyano-2-(pyridin-4-yl)ethyl)amino)-1-oxo-3-phenylpropan-2-yl)-1-methyl-1H-pyrazole-5-carboxamide (**55**)*

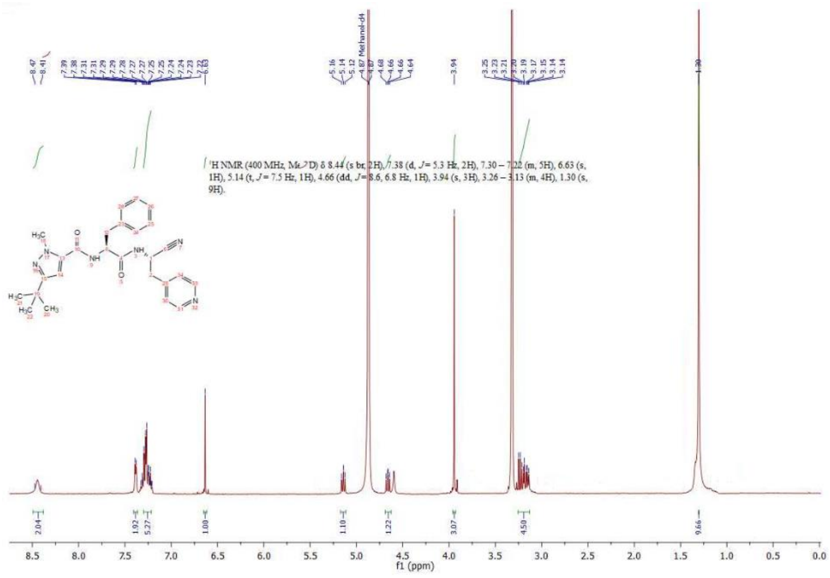

*Figure S49. ^{1}H-NMR (400 MHz, CD_3OD) of compound **55**.*

*Figure S50. ^{13}C NMR (100 MHz, CD_3OD) of compound **55**.*

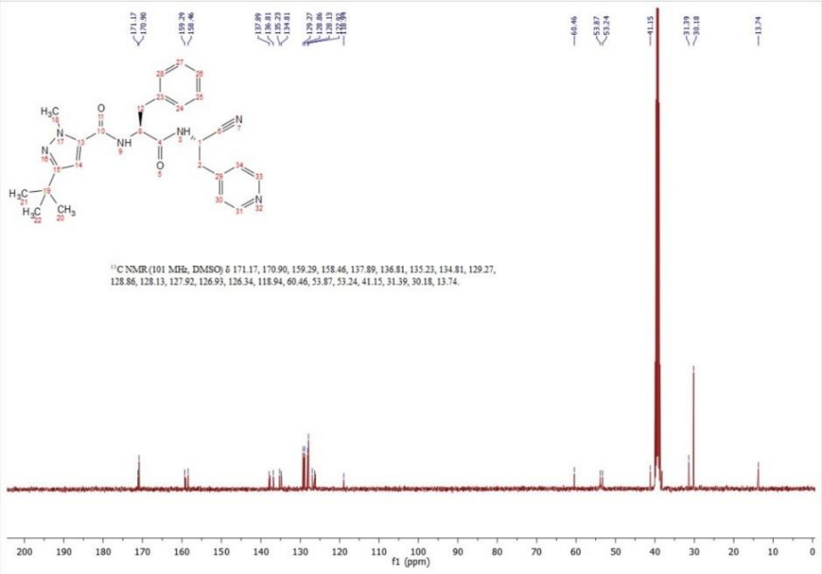

[illegible]

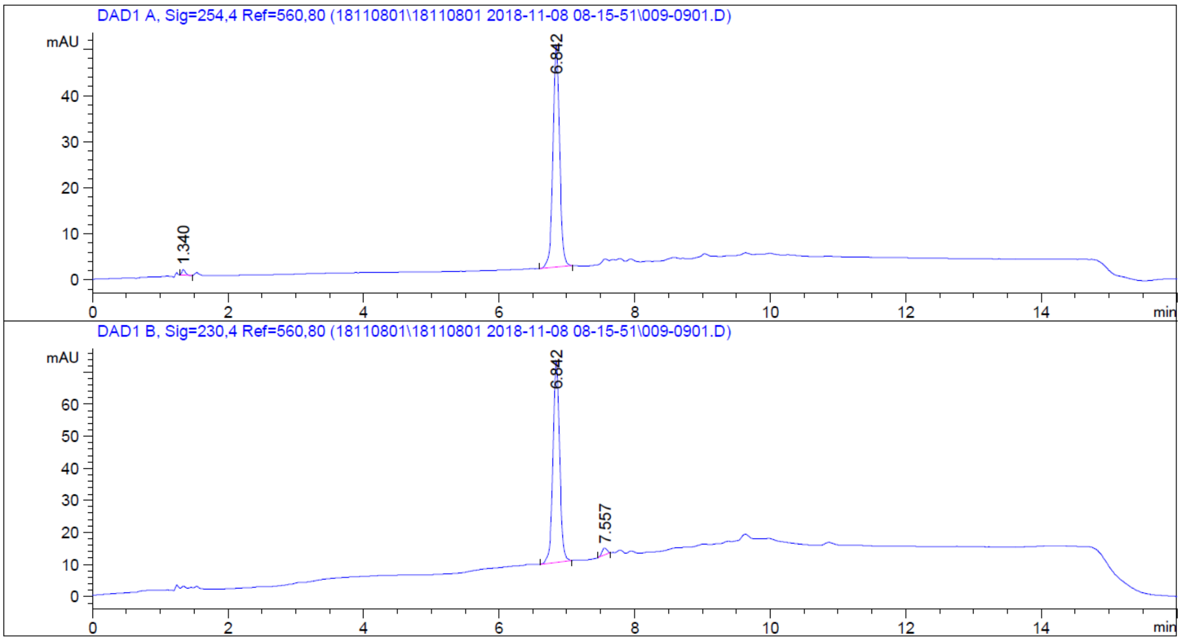

===

Area Percent Report

===

Sorted By : Signal
Multiplier: : 1.0000
Dilution: : 1.0000
Use Multiplier & Dilution Factor with ISTDs

Signal 1: DAD1 A, Sig=254,4 Ref=560,80

Peak #	RetTime [min]	Type	Width [min]	Area [mAU*s]	Height [mAU]	Area %
1	1.340	BB	0.0572	4.46968	1.20623	1.3366
2	6.842	BB	0.1057	329.93869	48.26745	98.6634
Totals :				334.40837	49.47368	

Figure S51. HPLC report of compound ***55****.*

*N-((S)-1-(((1R,2R)-2-(benzyloxy)-1-cyanopropyl)amino)-1-oxo-3-phenylpropan-2-yl)-3-(tert-butyl)-1-methyl-1H-pyrazole-5-carboxamide (**56**)*

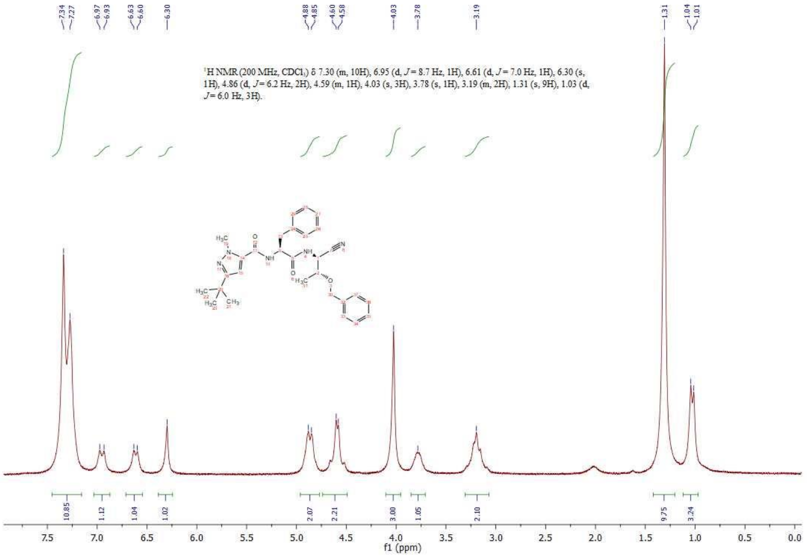

*Figure S52. ^{1}H-NMR (200 MHz, $CDCl_3$) for compound **56**.*

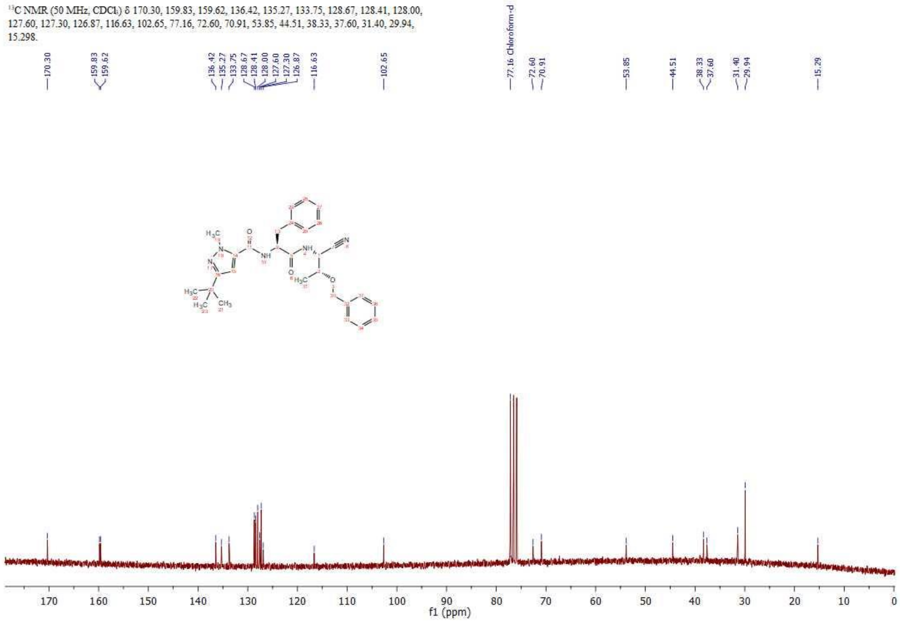

*Figure S53. ^{13}C NMR (50 MHz, $CDCl_3$) for compound **56**.*

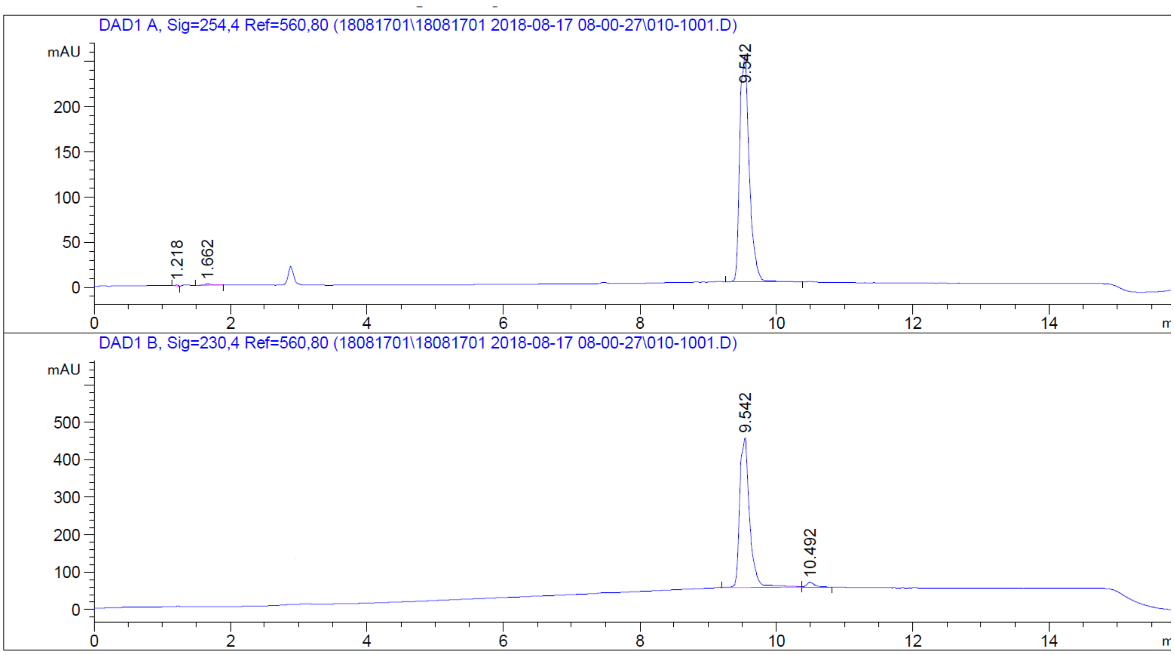

===
Area Percent Report
===

Sorted By : Signal
Multiplier: : 1.0000
Dilution: : 1.0000
Use Multiplier & Dilution Factor with ISTDs

Signal 1: DAD1 A, Sig=254,4 Ref=560,80

Peak #	RetTime [min]	Type	Width [min]	Area [mAU*s]	Height [mAU]	Area %
1	1.218	BB	0.0465	4.36268	1.47341	0.1770
2	1.662	BB	0.0865	8.65021	1.47317	0.3510
3	9.542	BB	0.1300	2451.75562	250.91428	99.4720
Totals :				2464.76850	253.86085	

*Figure S54. HPLC report of compound **56**.*

*N-((S)-1-(((1S,2S)-2-(benzyloxy)-1-cyanopropyl)amino)-1-oxo-3-phenylpropan-2-yl)-3-(tert-butyl)-1-methyl-1H-pyrazole-5-carboxamide (**57**).*

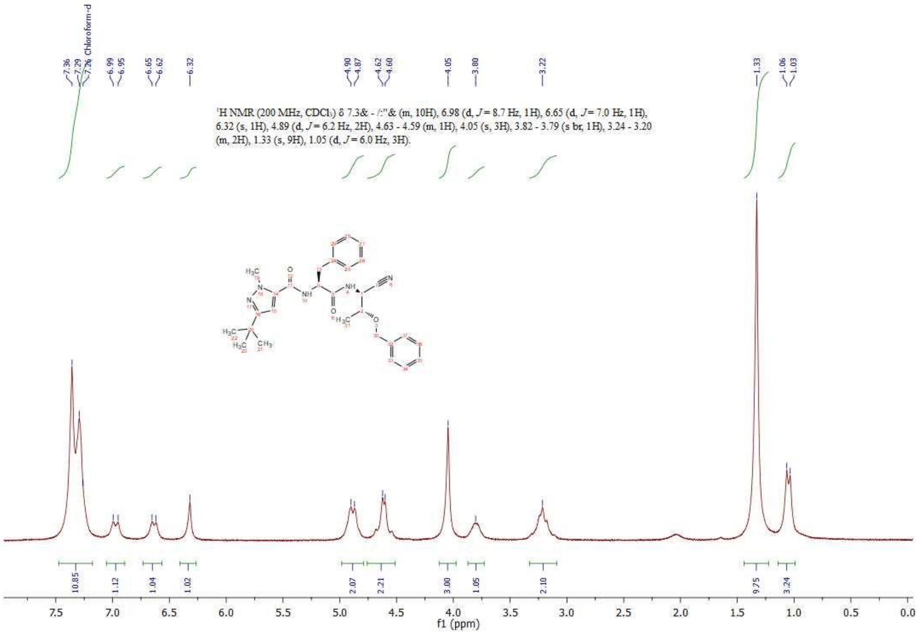

*Figure S55. ^{1}H-NMR (200 MHz, $CDCl_3$) of compound **57**.*

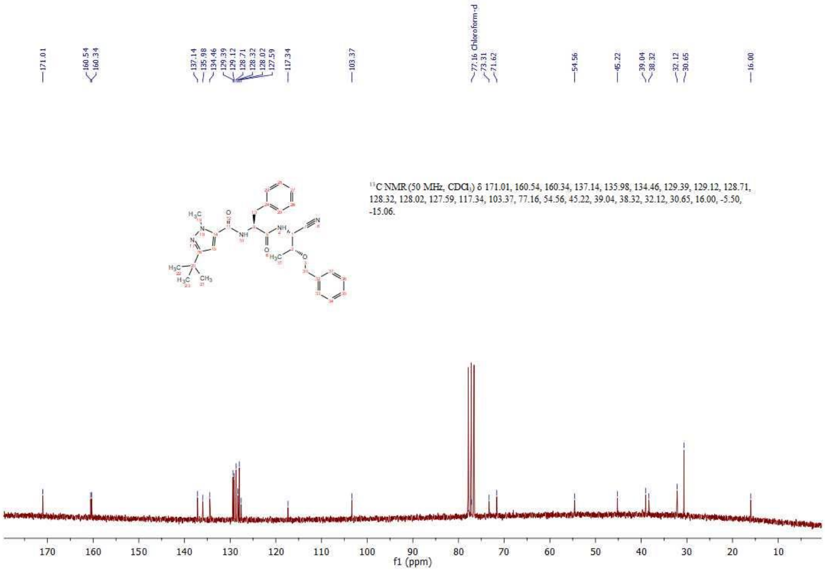

*Figure S56. ^{13}C NMR (50 MHz, $CDCl_3$) of compound **57**.*

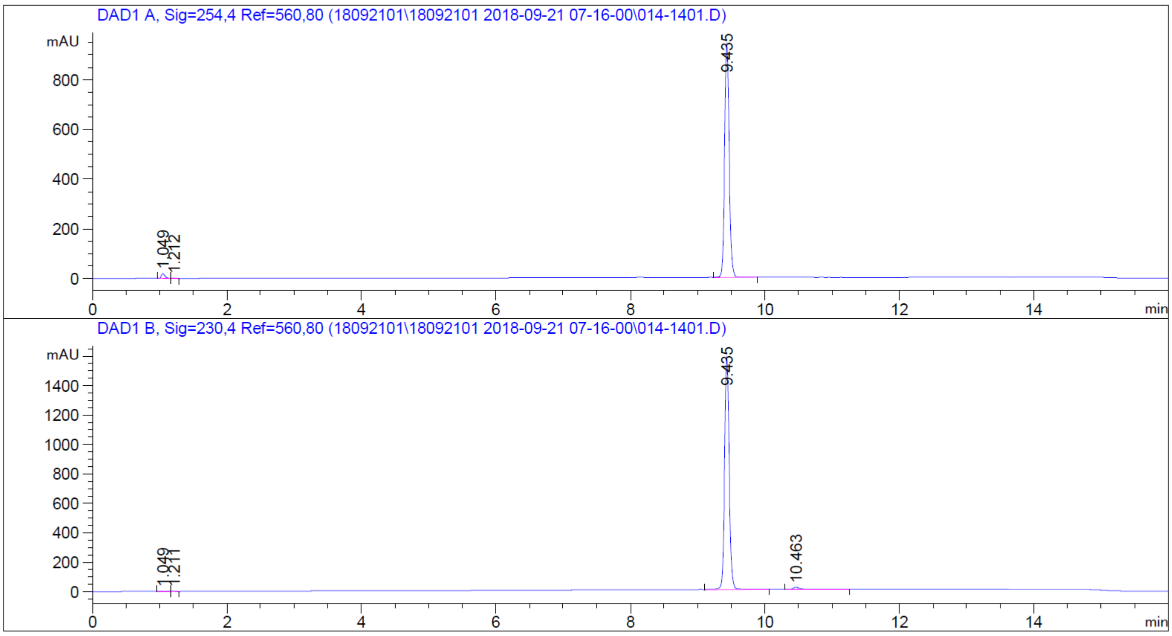

===

Area Percent Report

===

Sorted By : Signal
Multiplier: : 1.0000
Dilution: : 1.0000
Use Multiplier & Dilution Factor with ISTDs

Signal 1: DAD1 A, Sig=254,4 Ref=560,80

Peak #	RetTime [min]	Type	Width [min]	Area [mAU*s]	Height [mAU]	Area %
1	1.049	BV	0.0547	66.55391	18.17334	1.4939
2	1.212	VB	0.0542	7.26296	2.10746	0.1630
3	9.435	BB	0.0725	4381.07959	938.13513	98.3430

Totals : 4454.89646 958.41593

*Figure S57. HPLC report of compound **57**.*

*N-((S)-1-(((1R,2R)-2-(benzyloxy)-1-cyanopropyl)amino)-4-methyl-1-oxopentan-2-yl)-3-(tert-butyl)-1-methyl-1H-pyrazole-5-carboxamide (**58**)*

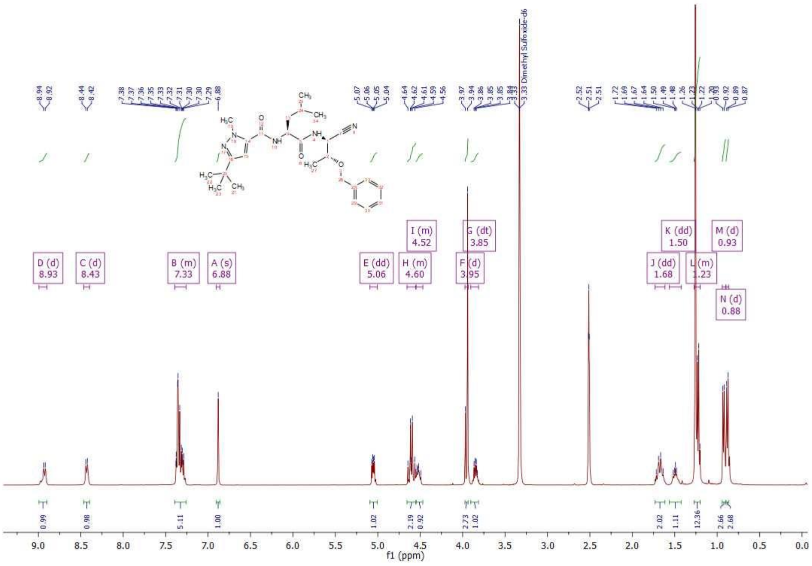

*Figure S58. ^{1}H-NMR (400 MHz, DMSO_d$_6$) of compound **58**.*

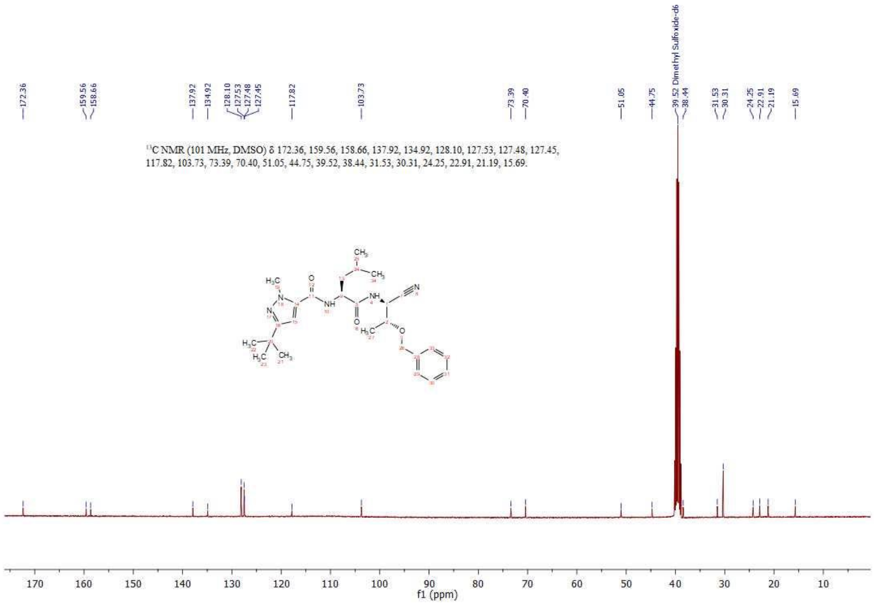

*Figure S59. ^{13}C NMR (100 MHz, DMSO_d$_6$) of compound **58**.*

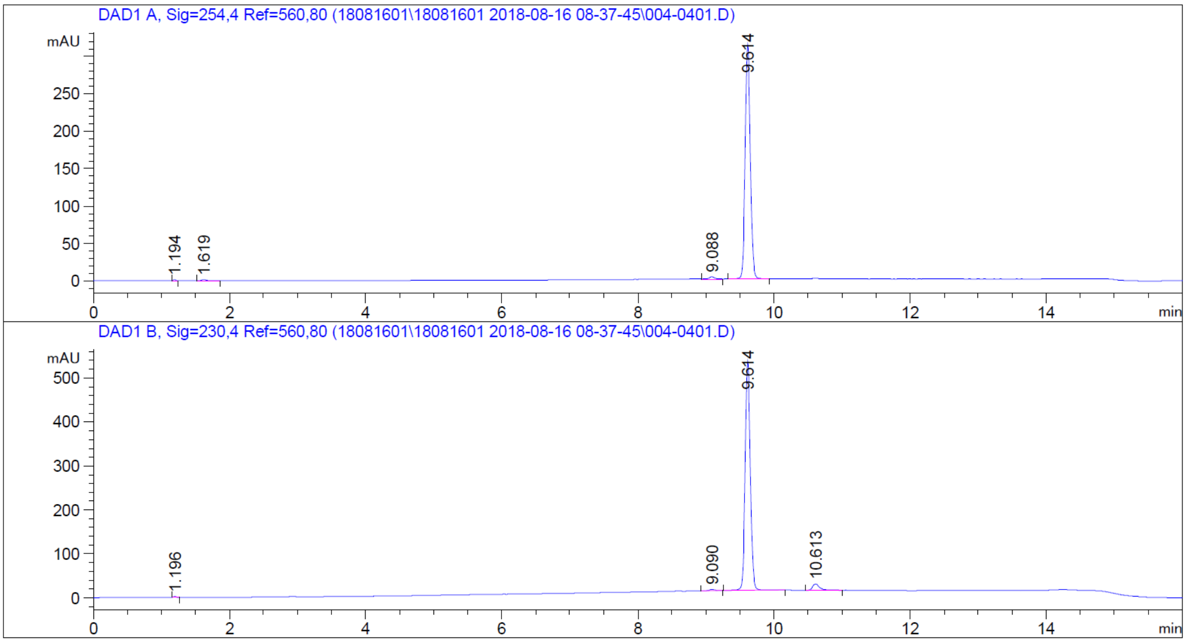

===
Area Percent Report
===

Sorted By : Signal
Multiplier: : 1.0000
Dilution: : 1.0000
Use Multiplier & Dilution Factor with ISTDs

Signal 1: DAD1 A, Sig=254,4 Ref=560,80

Peak #	RetTime [min]	Type	Width [min]	Area [mAU*s]	Height [mAU]	Area %
1	1.194	BB	0.0406	4.27551	1.74177	0.2568
2	1.619	BB	0.0748	7.89032	1.61939	0.4739
3	9.088	BB	0.0804	15.76121	2.94864	0.9467
4	9.614	BB	0.0811	1636.96045	312.73624	98.3226

Totals : 1664.88749 319.04603

*Figure S60. HPLC report of compound **58**.*

*N-((S)-1-(((1S,2S)-2-(benzyloxy)-1-cyanopropyl)amino)-4-methyl-1-oxopentan-2-yl)-3-(tert-butyl)-1-methyl-1H-pyrazole-5-carboxamide (**59**)*

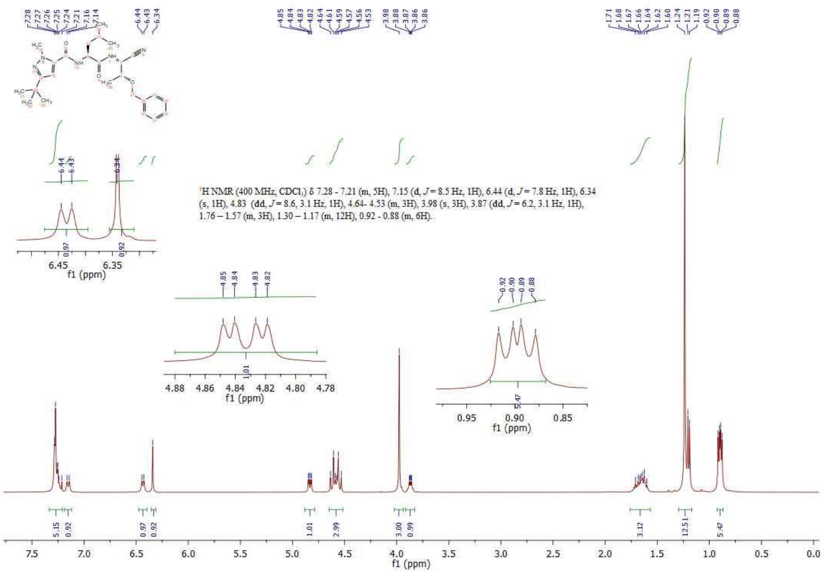

*Figure S61. ^{1}H-NMR (400 MHz, $CDCl_3$) of compound **59**.*

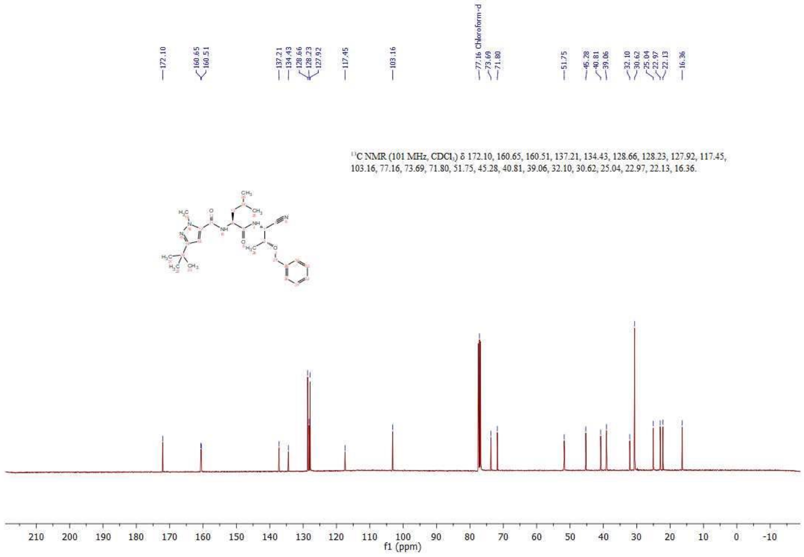

*Figure S62. ^{13}C NMR (100 MHz, $CDCl_3$) of compound **59**.*

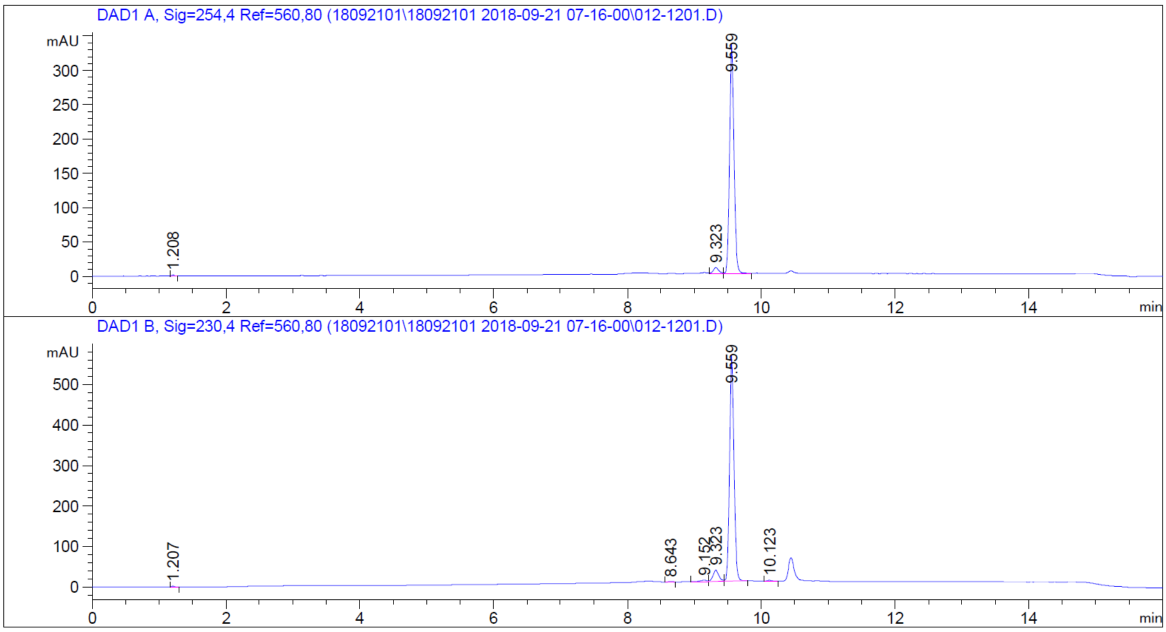

===

Area Percent Report

===

Sorted By : Signal
Multiplier: : 1.0000
Dilution: : 1.0000
Use Multiplier & Dilution Factor with ISTDs

Signal 1: DAD1 A, Sig=254,4 Ref=560,80

Peak #	RetTime [min]	Type	Width [min]	Area [mAU*s]	Height [mAU]	Area %
1	1.208	BB	0.0430	4.11630	1.54986	0.2594
2	9.323	BV	0.0822	43.75515	8.20472	2.7575
3	9.559	VB	0.0718	1538.88318	333.61386	96.9831

Totals : 1586.75463 343.36845

*Figure S63. HPLC report of compound **59**.*

*N-((S)-1-(((1R,2R)-2-(benzyloxy)-1-cyanopropyl)amino)-3-(3-chlorophenyl)-1-oxopropan-2-yl)-3-(tert-butyl)-1-methyl-1H-pyrazole-5-carboxamide (**60**)*

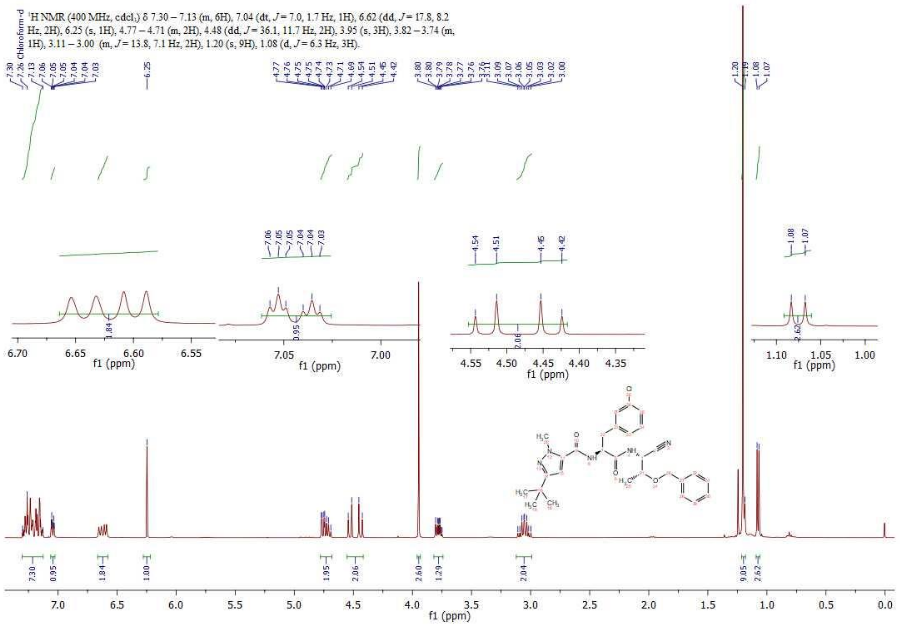

*Figure S64. ^{1}H NMR (400 MHz, $CDCl_3$) of compound **60**.*

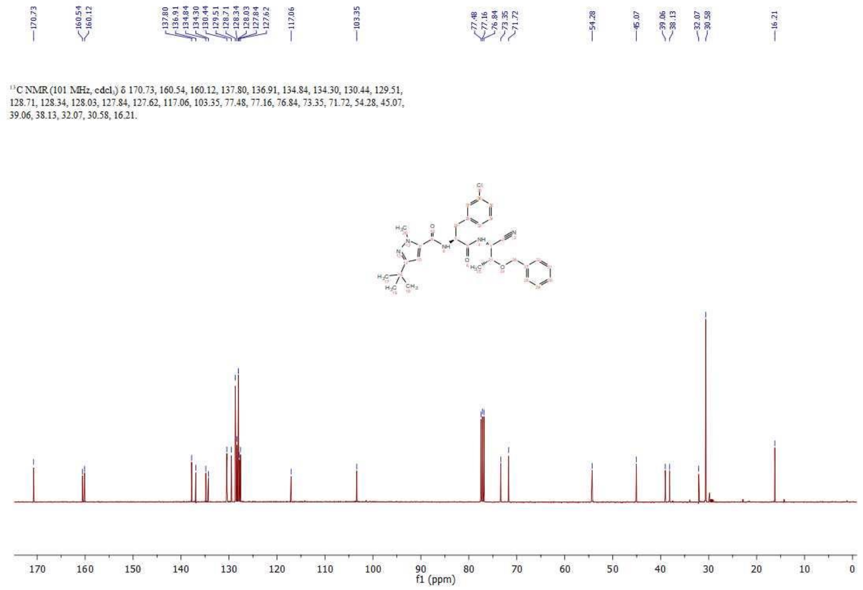

*Figure S65. ^{13}C NMR (100 MHz, $CDCl_3$) of compound **60**.*

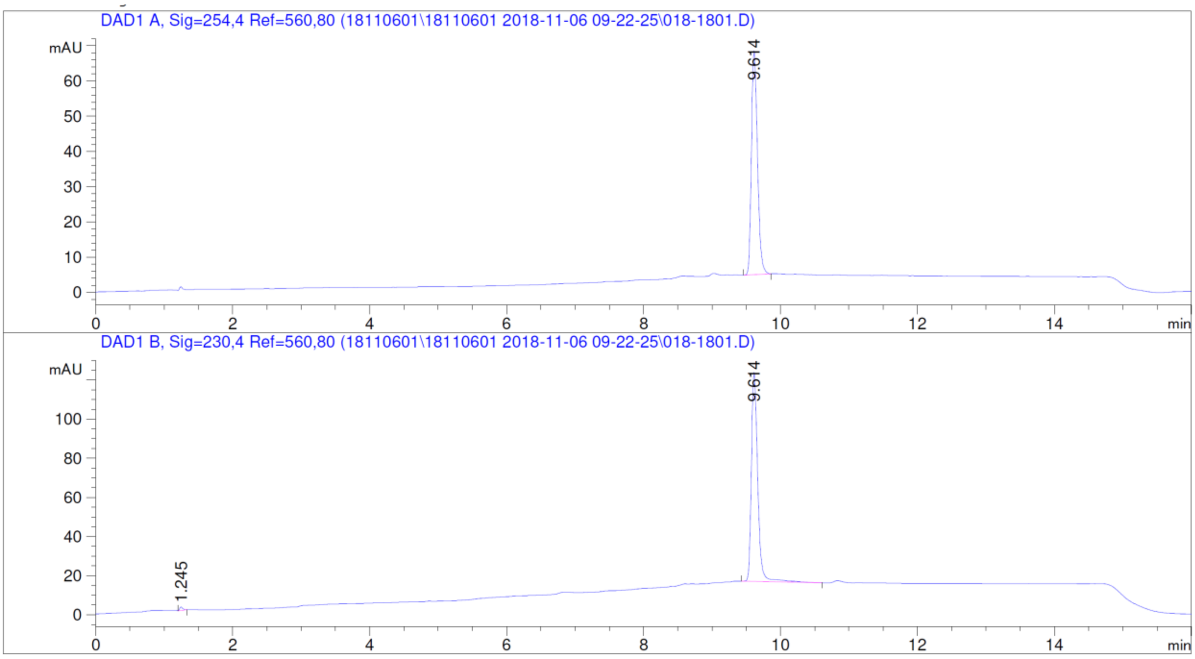


```
=====================================================================
                         Area Percent Report
=====================================================================

Sorted By              :      Signal
Multiplier:                    :      1.0000
Dilution:                      :      1.0000
Use Multiplier & Dilution Factor with ISTDs


Signal 1: DAD1 A, Sig=254,4 Ref=560,80
```

Figure S66. HPLC report of compound ***60.***

*3-(tert-butyl)-N-((S)-1-(((1R,2R)-1-cyano-2-hydroxypropyl)amino)-1-oxo-3-phenylpropan-2-yl)-1-methyl-1H-pyrazole-5-carboxamide (**65**)*

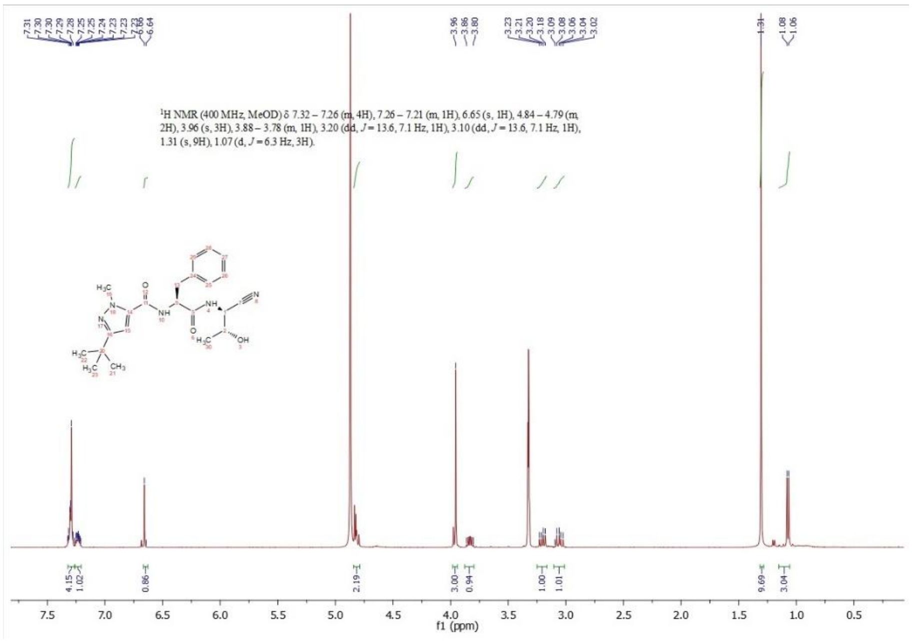

*Figure S67. ^{1}H NMR (400 MHz, $CDCl_3$) of compound **65**.*

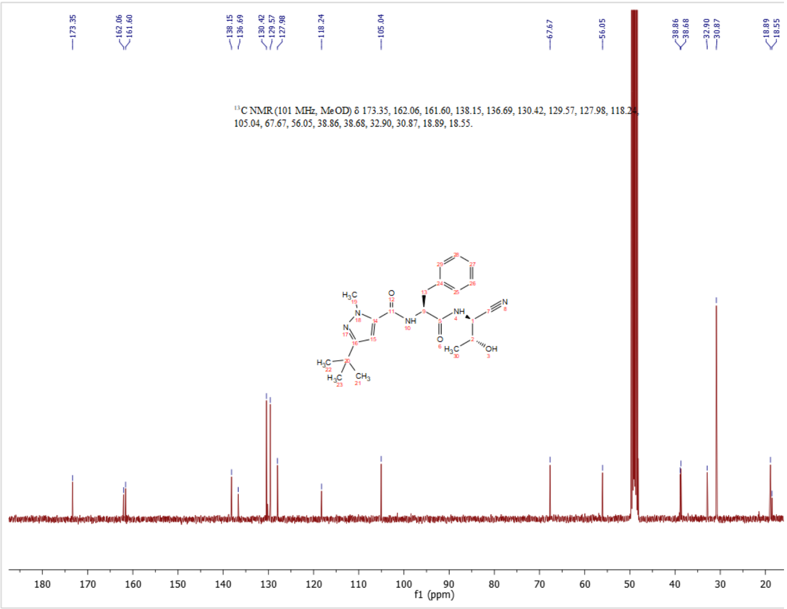

*Figure S68. ^{13}C NMR (100 MHz, $CDCl_3$) of compound **65**.*

Additional Info : Peak(s) manually integrated

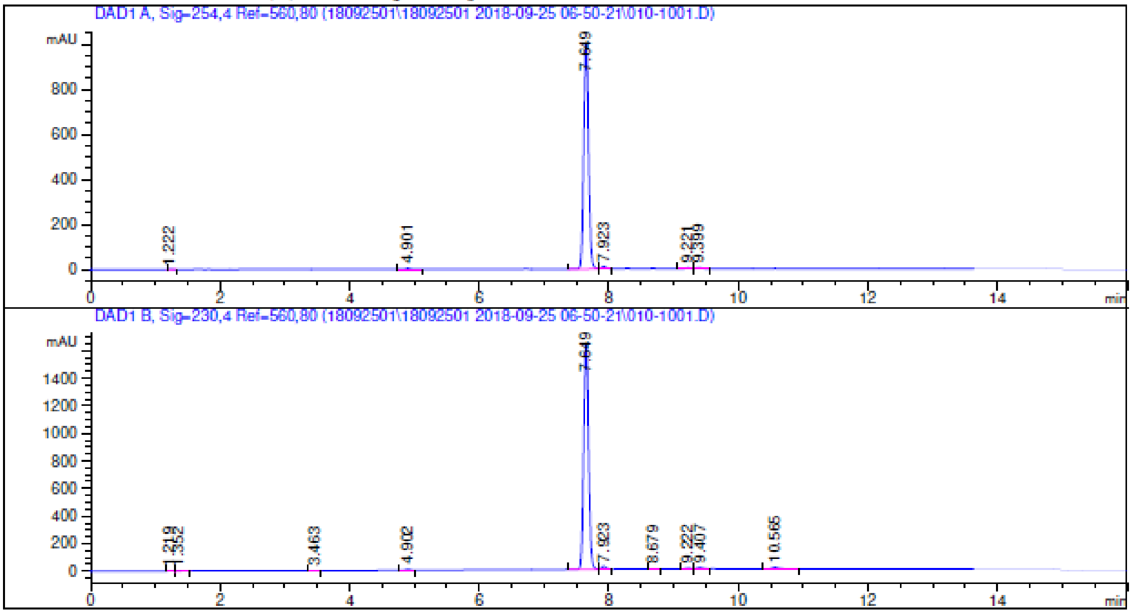


```
=====================================================================
                         Area Percent Report
=====================================================================

Sorted By             :      Signal
Multiplier:                   :      1.0000
Dilution:                     :      1.0000
Use Multiplier & Dilution Factor with ISTDs


Signal 1: DAD1 A, Sig=254,4 Ref=560,80

Peak RetTime Type  Width     Area      Height     Area
  #   [min]        [min]   [mAU*s]     [mAU]        %
----|-------|----|-------|----------|----------|--------|
   1   1.222 BB    0.0477    4.54292    1.40523   0.0873
   2   4.901 BB    0.0789   18.89323    3.74361   0.3632
   3   7.649 BV    0.0790 5085.84131 1005.46161  97.7664
   4   7.923 VB    0.0803   53.22422   10.29762   1.0231
   5   9.221 BV    0.0764   17.11441    3.41857   0.3290
   6   9.399 VB    0.0923   22.41987    3.82814   0.4310
```

Figure S69. HPLC report of compound ***65****.*

*3-(tert-butyl)-N-((S)-1-(((1S,2S)-1-cyano-2-hydroxypropyl)amino)-1-oxo-3-phenylpropan-2-yl)-1-methyl-1H-pyrazole-5-carboxamide (**66**)*

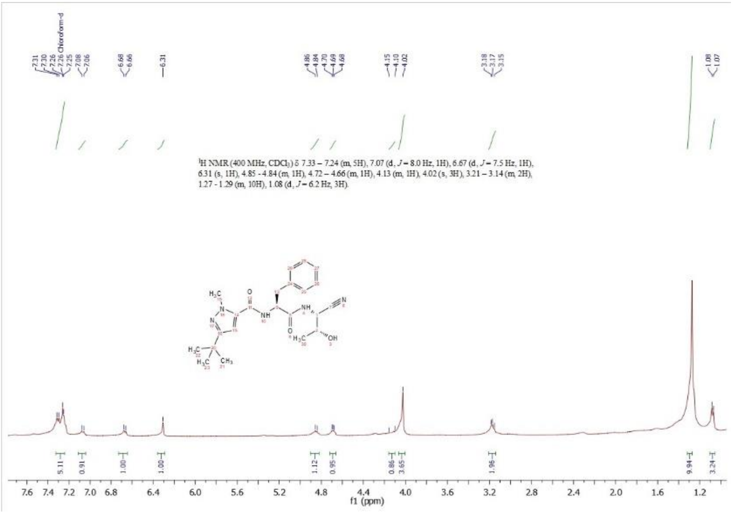

*Figure S70. ^{1}H NMR (400 MHz, $CDCl_3$) of compound **66**.*

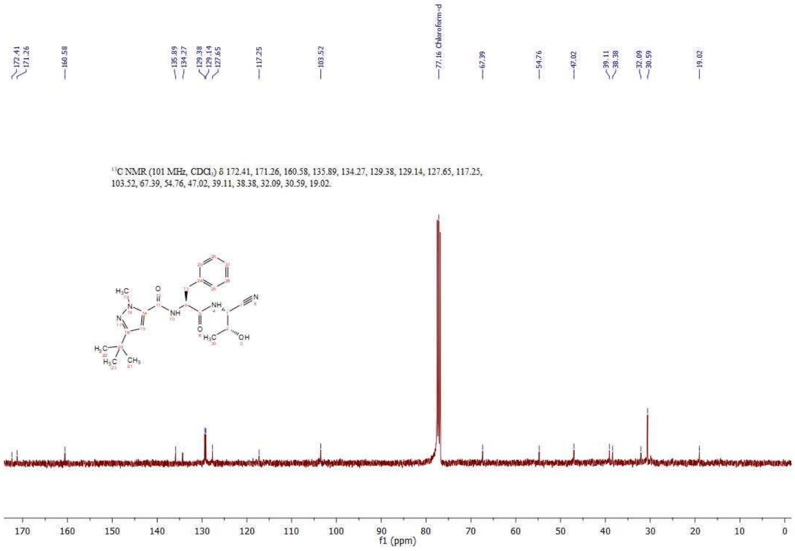

*Figure S71. ^{13}C NMR (100 MHz, $CDCl_3$) of compound **66**.*

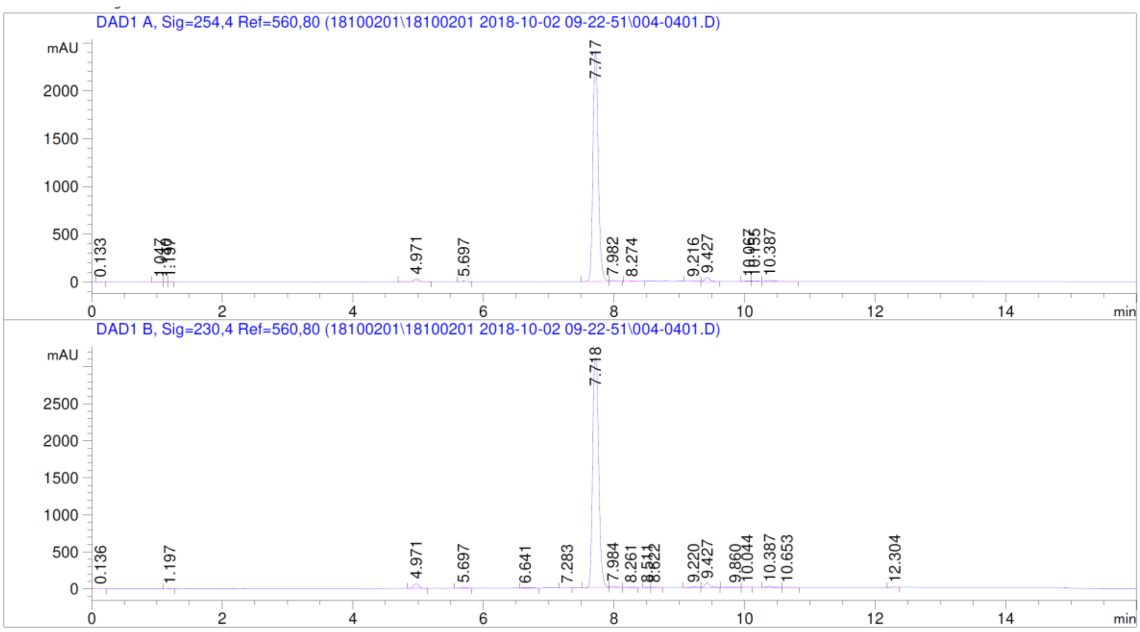

==

Area Percent Report

==

Sorted By : Signal
Multiplier: : 1.0000
Dilution: : 1.0000
Use Multiplier & Dilution Factor with ISTDs

Signal 1: DAD1 A, Sig=254,4 Ref=560,80

Peak #	RetTime [min]	Type	Width [min]	Area [mAU*s]	Height [mAU]	Area %
1	0.133	BB	0.0690	8.65173	1.77059	0.0607
2	1.047	BV	0.0583	6.67397	1.75814	0.0468
3	1.140	VV	0.0447	4.43405	1.49275	0.0311
4	1.197	VB	0.0437	7.14257	2.62673	0.0501
5	4.971	BB	0.0816	162.82047	30.83212	1.1418
6	5.697	BB	0.0797	23.71537	4.63831	0.1663
7	7.717	BV	0.0897	1.36200e4	2418.99512	95.5156
8	7.982	VB	0.0811	61.30469	10.98270	0.4299
9	8.274	BB	0.1138	23.80804	3.16470	0.1670
10	9.216	BV	0.0908	16.85124	2.77777	0.1182
11	9.427	VB	0.0738	203.93962	42.61785	1.4302
12	10.067	BV	0.0797	19.40185	3.55121	0.1361
13	10.155	VV	0.0868	23.16066	3.92686	0.1624

*Figure S72. HPLC report of compound **66***

*3-(tert-butyl)-N-((S)-1-(((1R,2R)-1-cyano-2-hydroxypropyl)amino)-4-methyl-1-oxopentan-2-yl)-1-methyl-1H-pyrazole-5-carboxamide (**67**)*

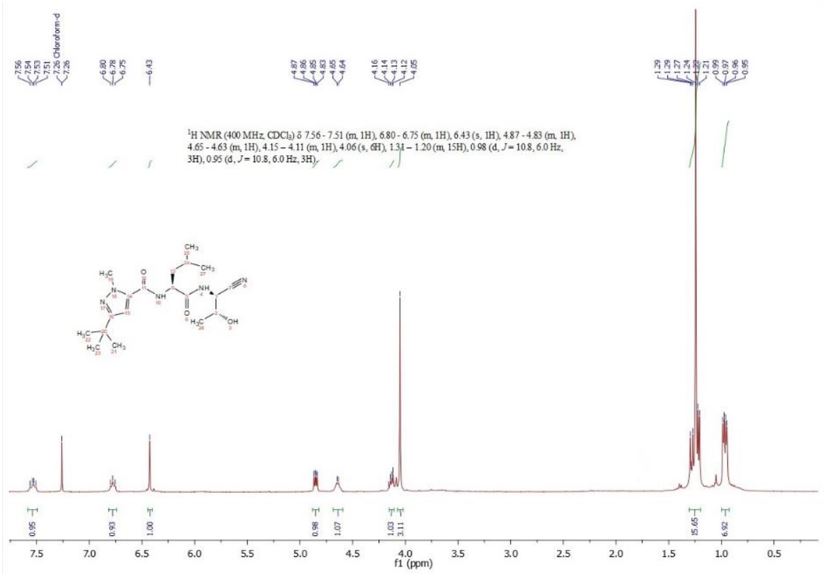

*Figure S73. ^{1}H NMR (400 MHz, $CDCl_3$) of compound **67**.*

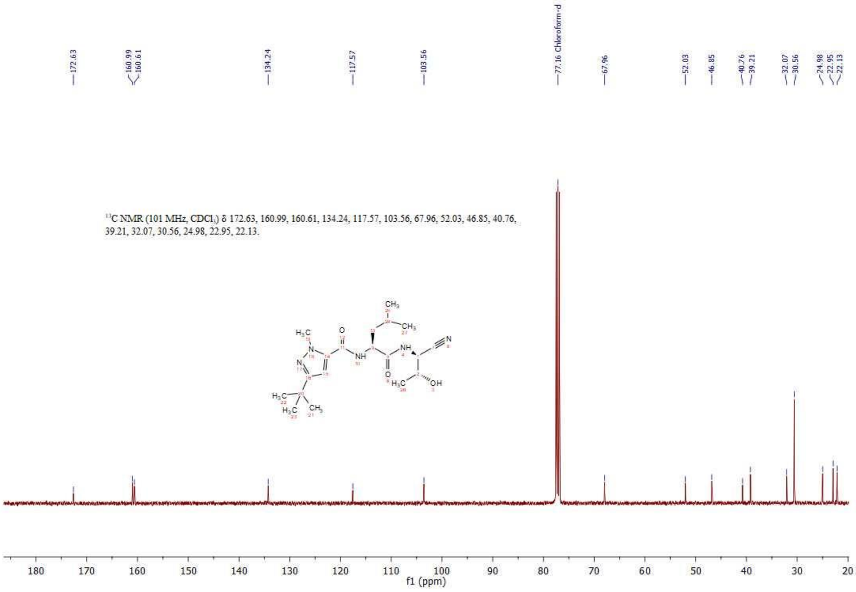

*Figure S74. ^{13}C NMR (100 MHz, $CDCl_3$) of compound **67**.*

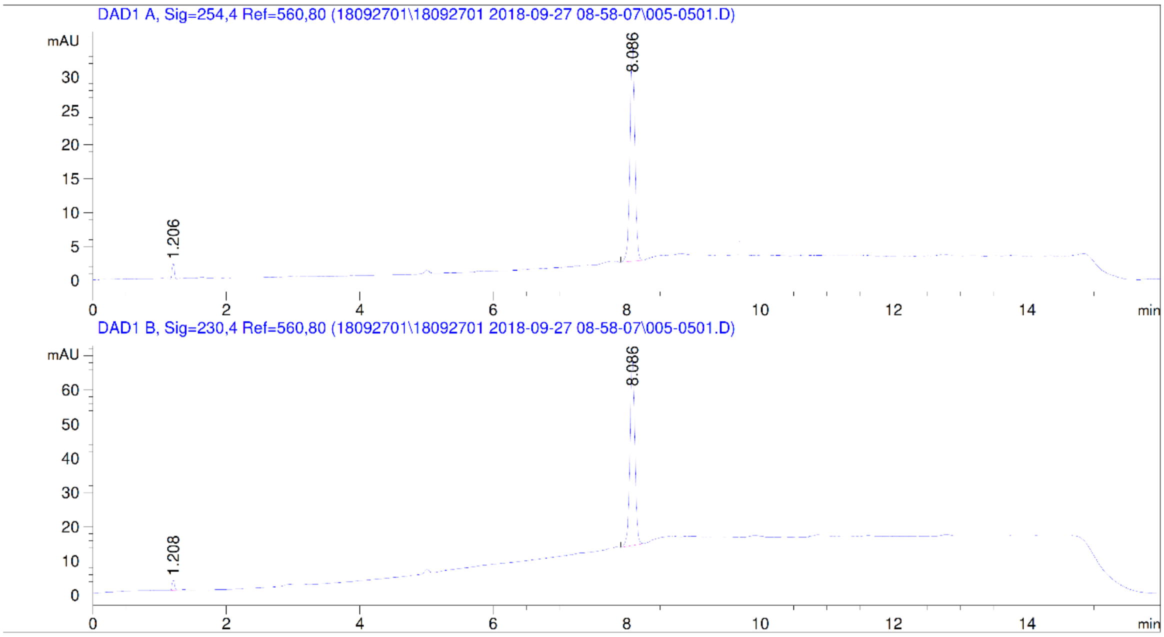

Area Percent Report

Sorted By : Signal
Multiplier: : 1.0000
Dilution: : 1.0000
Use Multiplier & Dilution Factor with ISTDs

Signal 1: DAD1 A, Sig=254,4 Ref=560,80

Peak #	RetTime [min]	Type	Width [min]	Area [mAU*s]	Height [mAU]	Area %
1	1.206	BB	0.0403	5.46627	2.25379	3.2423
2	8.086	BB	0.0773	163.12456	32.06937	96.7577

Totals : 168.59083 34.32317

Figure S75. HPLC report of compound ***67****.*

*3-(tert-butyl)-N-((S)-1-(((1S,2S)-1-cyano-2-hydroxypropyl)amino)-4-methyl-1-oxopentan-2-yl)-1-methyl-1H-pyrazole-5-carboxamide (**68**)*

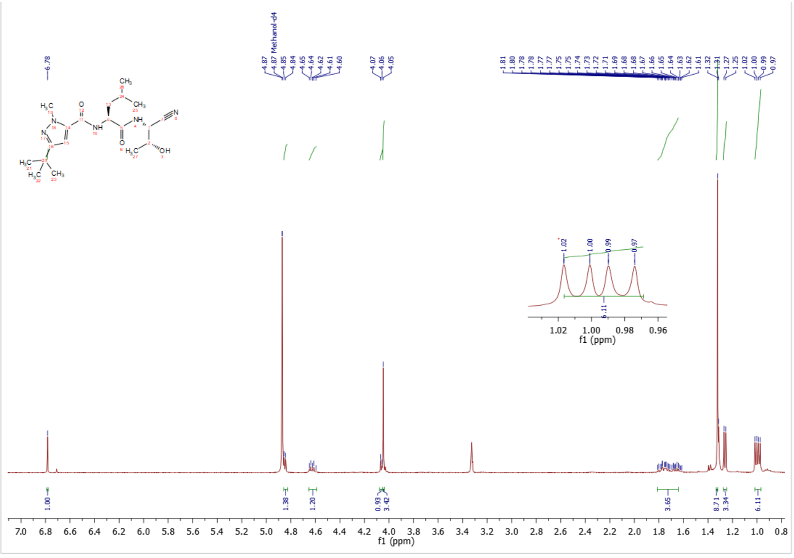

*Figure S76. ^{1}H NMR (400 MHz, CD_3OD) of compound **68**.*

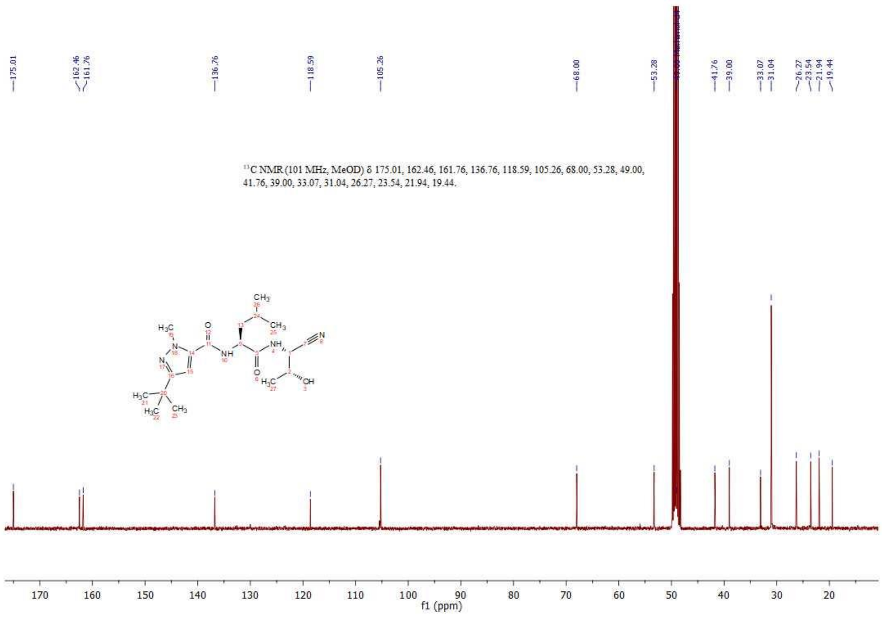

*Figure S77. ^{13}C NMR (100 MHz, CD_3OD) of compound **68**.*

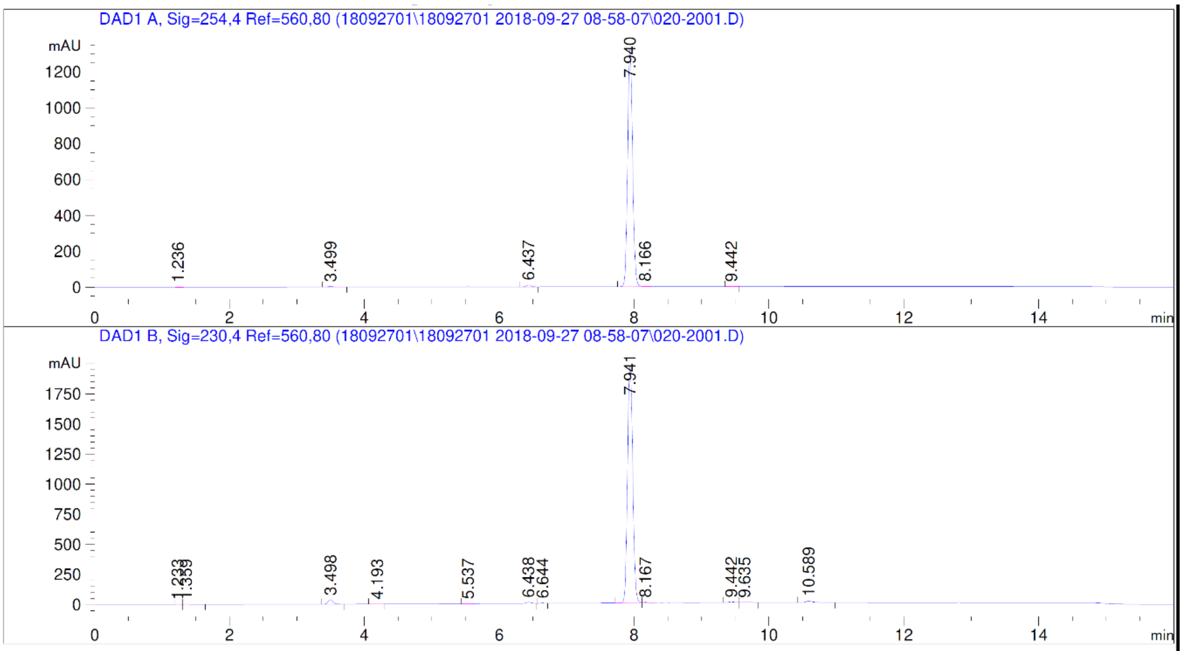

===

Area Percent Report

===

Sorted By : Signal
Multiplier: : 1.0000
Dilution: : 1.0000
Use Multiplier & Dilution Factor with ISTDs

Signal 1: DAD1 A, Sig=254,4 Ref=560,80

Peak #	RetTime [min]	Type	Width [min]	Area [mAU*s]	Height [mAU]	Area %
1	1.236	BB	0.0493	4.91261	1.53643	0.0718
2	3.499	BB	0.0782	22.10751	4.42866	0.3230
3	6.437	BB	0.0776	50.32943	10.18659	0.7352
4	7.940	BV	0.0814	6749.69727	1326.27588	98.6012
5	8.166	VB	0.0693	10.03590	2.19558	0.1466
6	9.442	BB	0.0740	8.37167	1.74480	0.1223

Figure S78. HPLC report of compound ***68****.*

*3-(tert-butyl)-N-((S)-3-(3-chlorophenyl)-1-(((1R,2R)-1-cyano-2-hydroxypropyl)amino)-1-oxopropan-2-yl)-1-methyl-1H-pyrazole-5-carboxamide (**69**)*

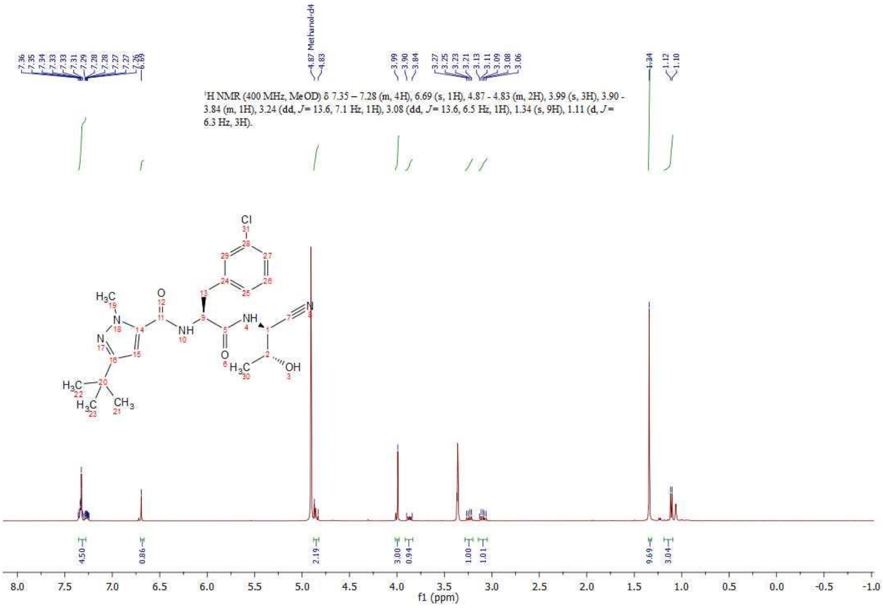

*Figure S79. ^{1}H NMR (400 MHz, CD_3OD) of compound **69**.*

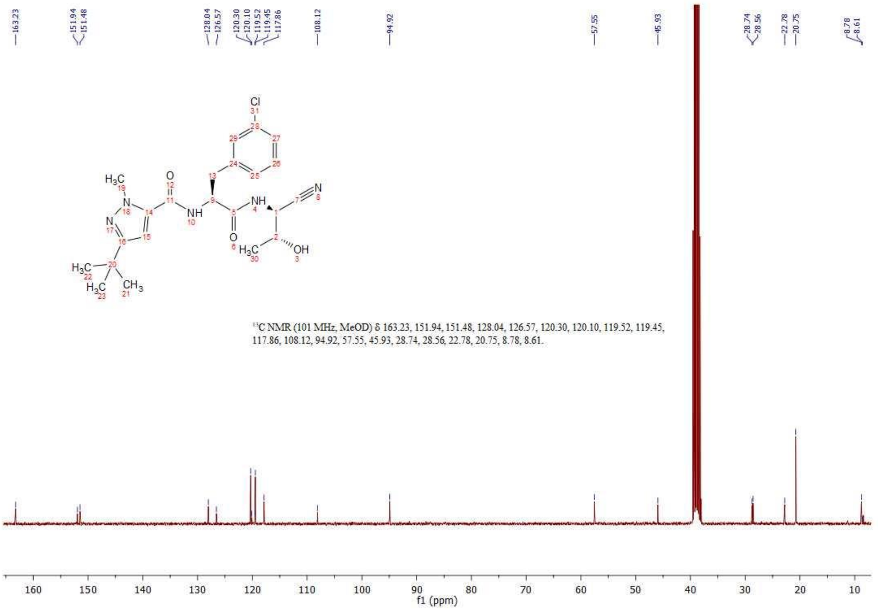

*Figure S80. ^{13}C NMR (100 MHz, CD_3OD) of compound **69**.*

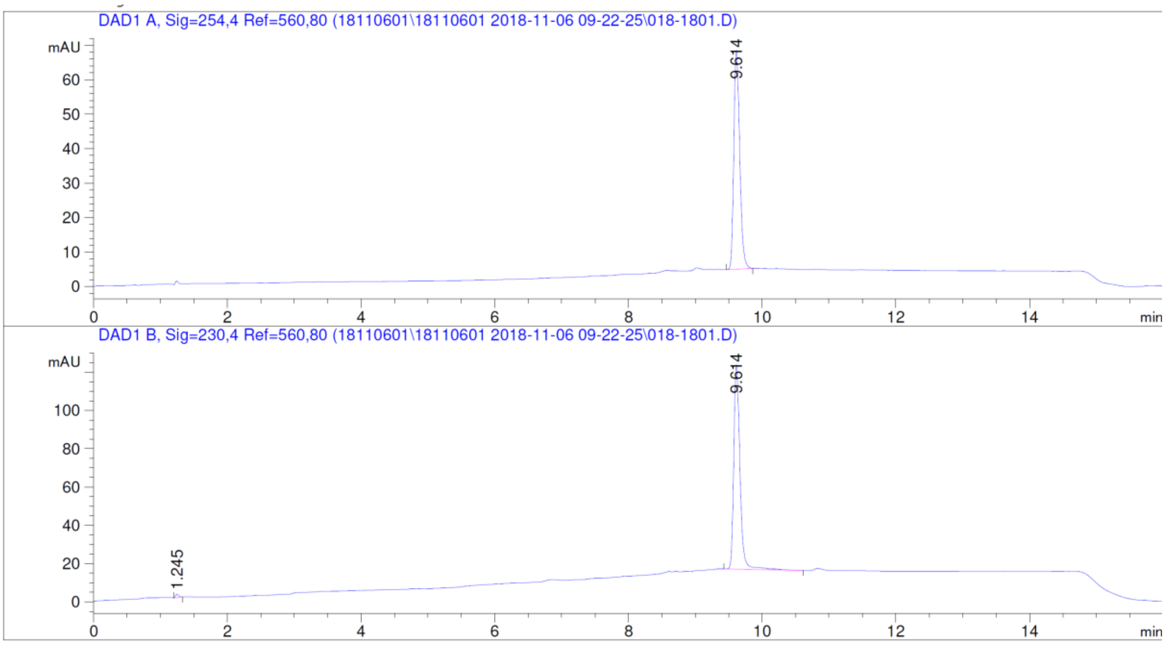

===

Area Percent Report

===

Sorted By : Signal
Multiplier: : 1.0000
Dilution: : 1.0000
Use Multiplier & Dilution Factor with ISTDs

Signal 1: DAD1 A, Sig=254,4 Ref=560,80

Peak #	RetTime [min]	Type	Width [min]	Area [mAU*s]	Height [mAU]	Area %
1	9.614	BB	0.0953	388.55350	63.56404	100.0000
Totals :				388.55350	63.56404	

Figure S81. HPLC report of compound ***69***

1. HPLC Analysis of Diasteroisomers

Three pair of diasteroisomers were used as a model to verify that the synthetic routes did not produce racemization. The absence of racemization was analyzed via HPLC instrument (Shimadzu LC) with a Diacel column (IC-chiralpak, 250 x 4.6 mm, 5 µm). Elution was carried out with the following gradient: water (solvent A), ACN (solvent B), 5 % B to 100 % B in 30 min, stop time 30 min, flow 0.5 ml/min.

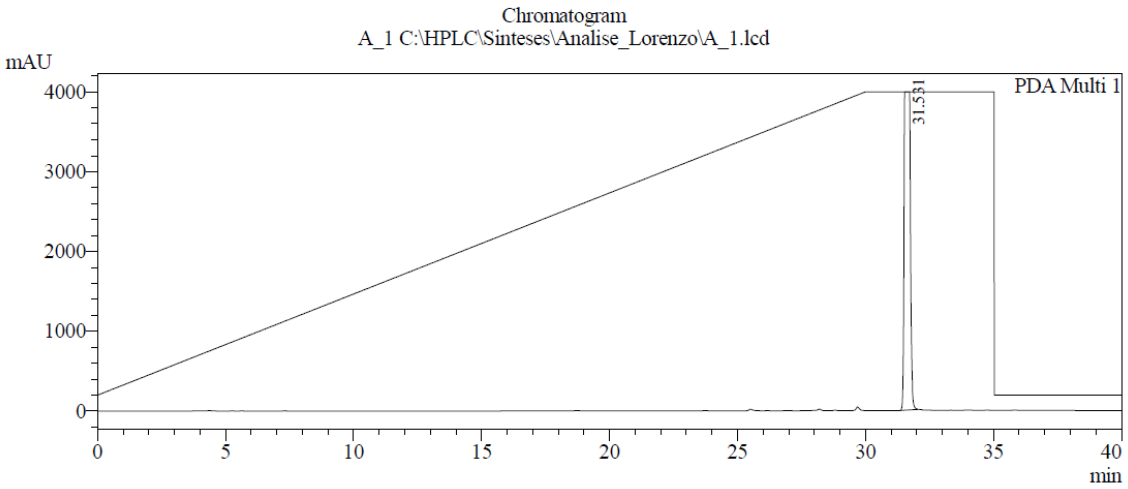

Figure S82. HPLC report with Diacel column of compound ***50****.*

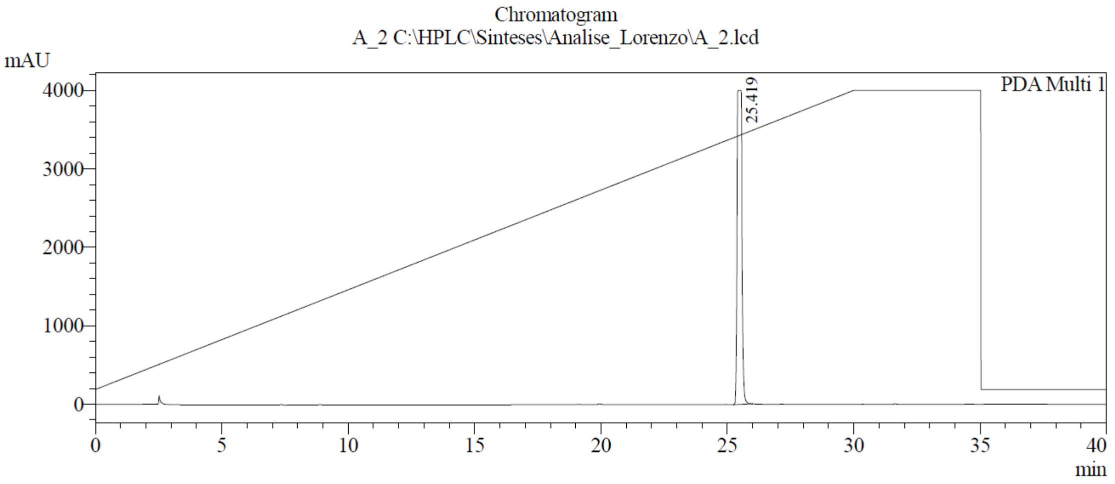

*Figure S83. HPLC report Diacel column of compound **51**.*

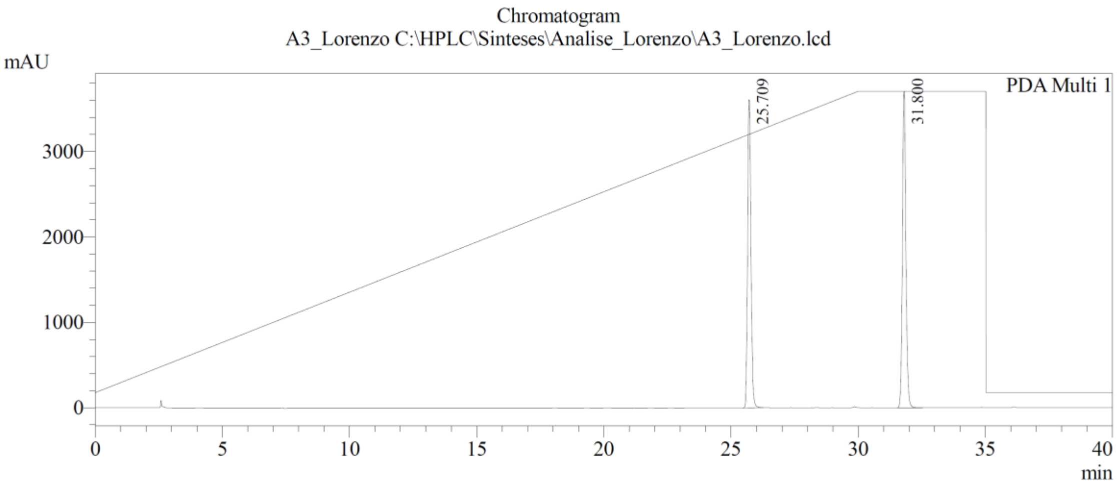

*Figure S84. HPLC report with Diacel column of a mixture of compounds **50** and **51**.*

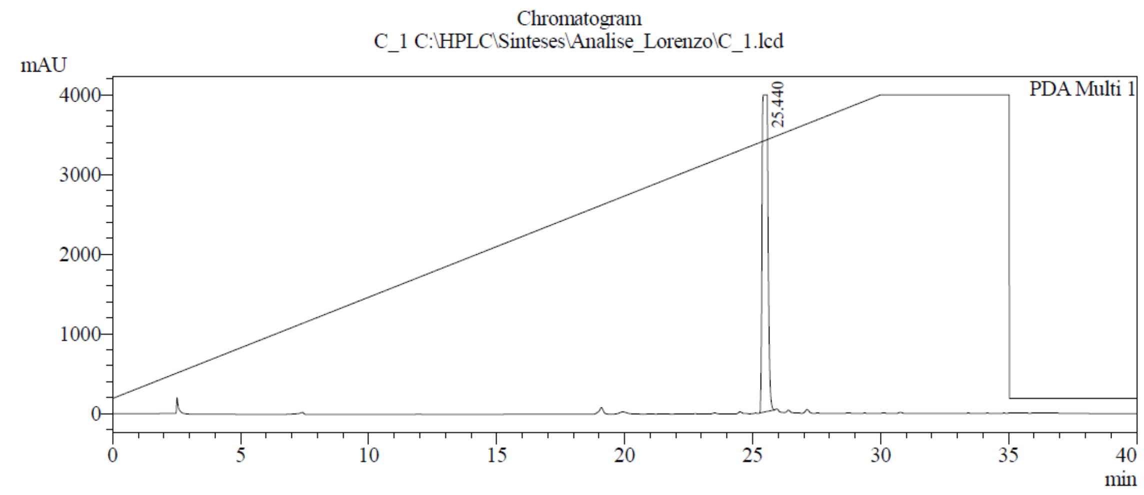

*Figure S85. HPLC report with Diacel column for compound **56**.*

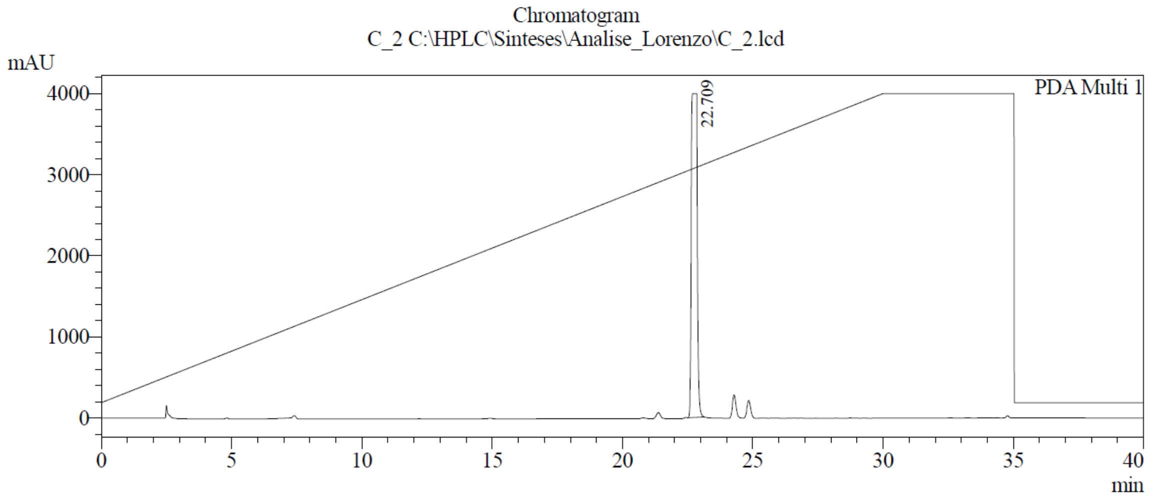

*Figure S86. HPLC report with Diacel column for compound **57**.*

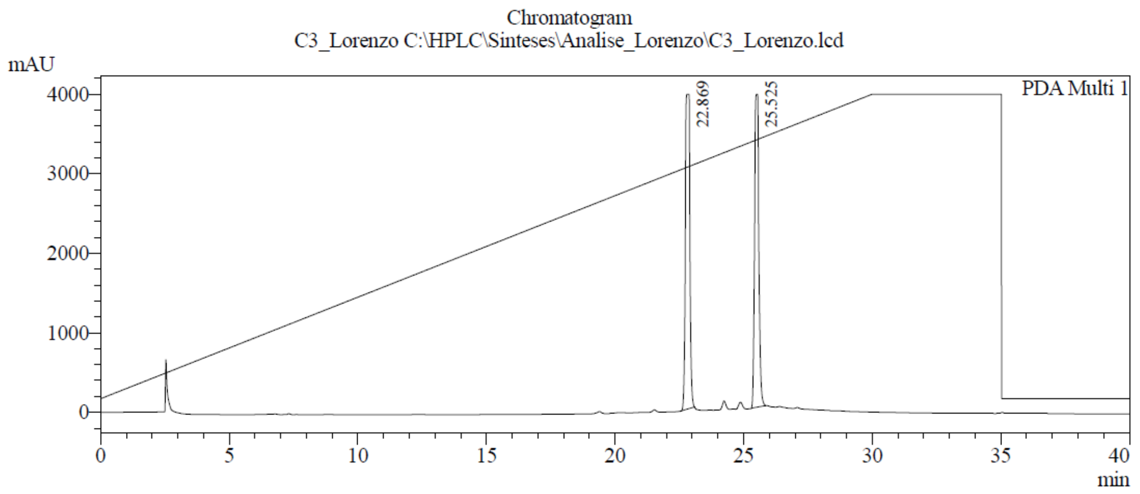

*Figure S87. HPLC report with Diacel column of a mixture of compound **56** and **57**.*

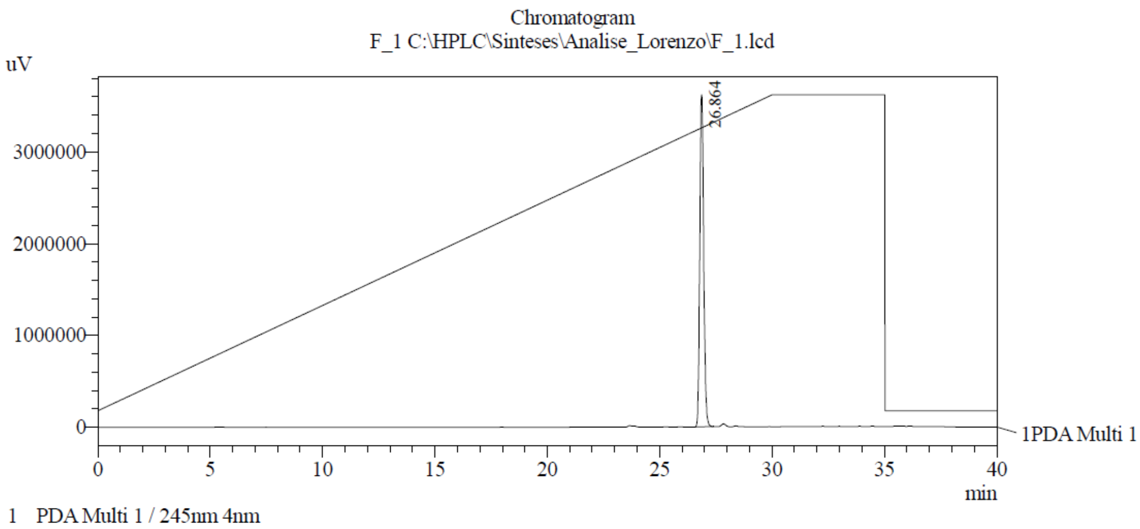

*Figure S88. HPLC report with Diacel column for compound **65**.*

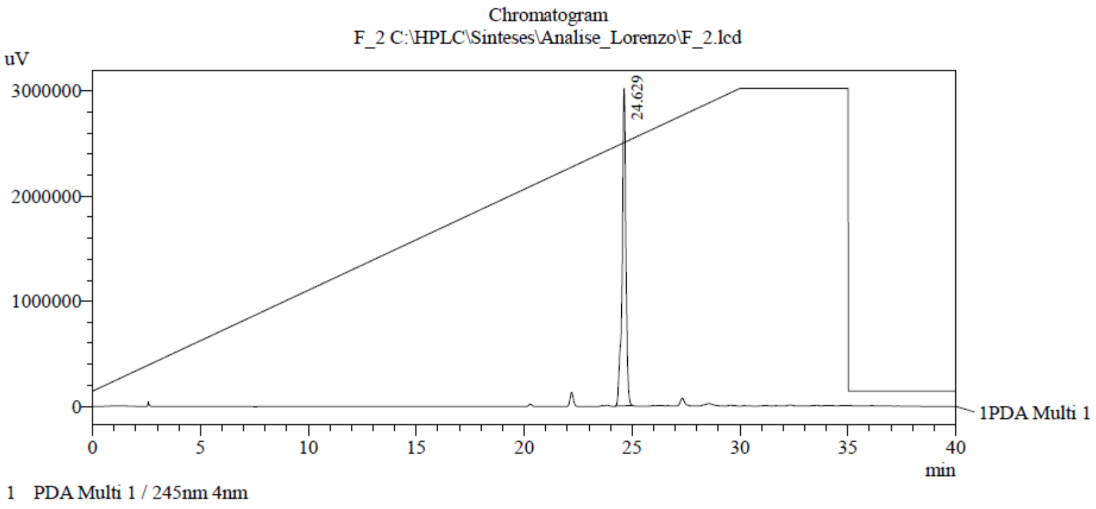

*Figure S89. HPLC report with Diacel column for compound **66**.*

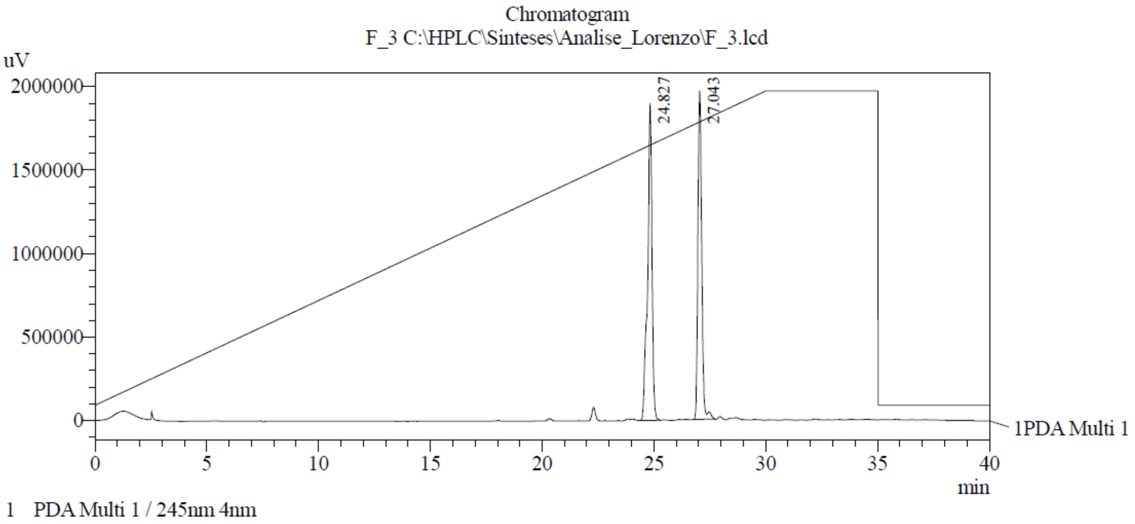

*Figure S90. HPLC report with Diacel column of a mixture of compound **65** and **66**.*
