# Supplementary material for: Mapping the S1 and S1’ subsites of cysteine proteases with new dipeptidyl nitrile inhibitors as trypanocidal agents
Source: PLoS Negl Trop Dis. 2020 Mar 12;14(3):e0007755. doi: 10.1371/journal.pntd.0007755 (PMC7067379; doi:10.1371/journal.pntd.0007755)
Supplement: S1 Table — Number identification, Nequimed number, biological data for trypanocidal activity (EC50) and citotoxicity (CC50). (PDF) [file pntd.0007755.s004.pdf]

. Number identification, Nequimed number, Biological data for trypanocidal activity (EC_{50}) and citotoxicity (CC_{50}).

Cmpd.	pEC_{50}	$EC_{50}^{T. cruzi}$(μM)	CC_{50}(μM)	SI
Neq0570 (**6**)	< 4	> 100	> 100	n.a.
Neq0865 (**7**)	< 4	> 100	4.0	n.a.
Neq0543 (**8**)	< 4	> 100	> 100	n.a.
Neq0533.1 (**9**)	4.1	71.8	> 100	n.a.
Neq0945 (**10**)	4.2	67.6	> 100	n.a.
Neq0569 (**11**)	< 4	> 100	> 100	n.a.
Neq0956 (**12**)	4.5	32.7	> 100	n.a.
Neq0719 (**13**)	< 4	> 100	> 100	n.a.
Neq0775		> 100	> 100	n.a.

(**14**)	< 4			
Neq0708 (**15**)	4.1	77.3	> 100	n.a.
Neq0777 (**16**)	4.3	47.3	> 100	n.a.
Neq0955 (**17**)	< 4	> 100	> 100	n.a.
Neq0958 (**18**)	< 4	> 100	> 100	n.a.
Neq0957 (**19**)	< 4	> 100	> 100	n.a.
Neq0937 (**50**)	4.2	63.5	83.1	1.3
Neq0866.1 (**51**)	5.1	8.6	42.34	4.9
Neq0940 (**52**)	5.4	4.1	97.9	23.9
Neq0952 (**53**)	4.6	26.0	131.9	5.1

Neq0953 (**54**)	4.8	15.8	44.7	2.8
Neq0954 (**55**)	< 4	> 100	> 100	n.a.
Neq0921 (**56**)	< 4	> 100	> 100	n.a.
Neq0938 (**57**)	5.4	4.3	141.2	32.5
Neq0922 (**58**)	< 4	> 100	> 100	n.a.
Neq0939 (**59**)	< 4	> 100	> 100	n.a.
Neq0877 (**60**)	5.3	4.9	124.7	25.3
Neq0941 (**65**)	4.1	70.8	> 100	n.a.
Neq0943 (**66**)	4.5	30.0	> 100	n.a.
Neq0942		24.4	> 100	n.a.

(**67**)	4.6			
Neq0944<br><br>(**68**)	4.8	16.3	> 100	n.a.
Neq0948<br><br>(**69**)	4.5	30.5	> 100	n.a.
Benznidazole	5.4	4.3	> 100	n.a.

Benznidazole was used as positive control. DMSO was used to dissolve compounds and as a negative control. Maximum concentration used 250 µM. n.a. = not applicable. All experiments were at least performed in duplicate.
